# High performance white blood cell classification using ensemble CNNs with Monte Carlo dropout and conformal prediction for clinical uncertainty quantification

**DOI:** 10.3389/fmed.2026.1959061

**Published:** 2026-10-12

**Authors:** Shahid Mehmood, Abeer A. K. Alharbi, Mona Alkhattabi, Muhammad Adnan Khan, Muhammad Amir Khan

**Affiliations:** 1 Department of Computer Science, Bahria University, Lahore, Pakistan; 2 Faculty of Computing, Riphah International University, Islamabad, Pakistan; 3 Information Systems Department, College of Computer and Information Sciences, Imam Mohammad Ibn Saud Islamic University (IMSIU), Riyadh, Saudi Arabia; 4 Department of Software, Faculty of Artificial Intelligence and Software, Gachon University, Seongnam-si, Republic of Korea; 5 Faculty of Computer and Mathematical Sciences, Universiti Teknologi MARA, Shah Alam, Selangor, Malaysia

**Keywords:** conformal prediction, ensemble learning, interpretable AI, medical image analysis, uncertainty quantification, white blood cell classification

## Abstract

Automated classification of white blood cell (WBC) images may support cell-morphology workflows, but high accuracy alone does not establish clinical readiness. We developed an Uncertainty-Guided Deep Ensemble (UGDE) combining MobileNetV3-Large, EfficientNet-B0, and ShuffleNetV2 with Monte Carlo dropout, temperature scaling, split conformal prediction, and Grad-CAM. The primary dataset contained 16,027 single-cell images from 78 patients (33 with leukemia and 45 without leukemia). All stated development partitions were grouped by patient, and augmentation was restricted to the proper training subset. On the held-out internal test set (3,205 images from 16 patients), UGDE achieved approximately 99% image-level accuracy and macro-F1 of 0.99. Expected calibration error was 2.3% before and 0.8% after temperature scaling. Internal empirical conformal coverage was 94.2%, 89.7%, and 79.7% at nominal coverage levels of 95%, 90%, and 80%, respectively; these slight shortfalls are reported without claiming that the targets were met. External evaluation used 2000 Raabin-WBC images after mapping the nine model outputs to five shared leukocyte categories and yielded 94% descriptive accuracy. Grad-CAM examples were used as *post-hoc* visualizations and were not interpreted as causal explanations. These retrospective results support further evaluation of uncertainty-aware WBC image classification, but they do not demonstrate performance on whole slides, automated differential counts, prospective patients, or clinical workflows.

## 1 Introduction

Accurate classification of white blood cells in peripheral blood smears is crucial for diagnosing hematological conditions, including leukemias. Manual microscopic examination by experts is the gold standard, but it is labor-intensive and subject to inter-observer variability (1). Automated image-based WBC classification using deep learning has shown promise in performing differential counts rapidly and objectively. In recent years, convolutional neural networks (CNNs) have achieved high accuracy (>98%) in classifying normal leukocyte types (neutrophils, lymphocytes, monocytes, eosinophils, basophils) from images (2–12). However, fewer studies address the distinction between pathological cells, such as leukemic blasts, and normal cells, especially on large, high-quality datasets. Bodzás et al. (1) addressed this issue by releasing a high-resolution WBC image dataset that encompasses both normal cells and blasts. This dataset enables the training of robust models that recognize subtle morphological differences (e.g., the larger nuclei and finer chromatin of blasts versus those of normal lymphocytes).

While high accuracy is desirable, clinical deployment of such models also demands reliability and transparency. Traditional deep learning models function as “black-boxes,” making decisions that are opaque and difficult to interpret (13–18). This lack of transparency poses a barrier to clinical adoption, as clinicians may be reluctant to trust AI systems without understanding their reasoning (19). Two key aspects are often lacking in prior studies: (1) Uncertainty quantification, the ability for the model to know when it is unsure. In medical diagnosis, an AI system should flag ambiguous cases for human review rather than output overconfident, incorrect predictions. Traditional CNNs output point estimates without uncertainty; recent Bayesian deep learning techniques address this by approximating probability distributions over predictions (20). (2) Interpretability, explaining why the model made a specific prediction. Black-box predictions are challenging to trust in medicine; thus, methods like Grad-CAM have been employed to highlight image regions that are influential to a CNN’s decision, providing visual validation that the model focuses on medically relevant features (21, 22).

To meet these needs, we propose an uncertainty-guided deep ensemble (UGDE) for WBC classification. We integrate three efficient CNN models and leverage Monte Carlo dropout during inference to quantify predictive uncertainty. We also apply Grad-CAM to interpret model decisions by producing heatmaps over cell images. To our knowledge, this is one of the first studies uniting deep ensembles, Bayesian uncertainty estimation, and explainable AI in the context of white blood cell image classification.

### 1.1 Methodological novelty and contributions

Unlike prior white blood cell (WBC) classification studies that primarily focus on maximizing predictive accuracy, this work introduces a reliability-centered framework that systematically integrates uncertainty modeling, calibration analysis, and interpretability within a unified clinical AI system. The proposed Uncertainty-Guided Deep Ensemble (UGDE) represents, to the best of our knowledge, among the first framework in WBC image classification that simultaneously combines heterogeneous lightweight CNN ensembling, Monte Carlo dropout–based epistemic uncertainty estimation, temperature-based probability calibration, inductive conformal prediction for distribution-free coverage guarantees, and Grad-CAM–based morphological interpretability. While each of these components has been studied independently in broader machine learning literature, their principled integration into a single deployable hematology classification pipeline constitutes a substantive methodological advancement beyond backbone reuse.

A key novelty of this study is the incorporation of inductive conformal prediction (ICP). Unlike softmax confidence or entropy-based uncertainty, conformal prediction ensures that the true label is contained within the prediction set with a user-defined probability, independent of distributional assumptions. To our knowledge, this is among the first WBC classification study to report empirical coverage analysis, prediction set size behavior, and risk–coverage trade-offs under conformal inference.

Furthermore, the proposed framework emphasizes calibration and uncertainty evaluation as first-class performance criteria. In contrast to most previous WBC classification works that report only accuracy, precision, and recall, we conduct comprehensive reliability analysis including Expected Calibration Error (ECE), reliability diagrams, entropy-based uncertainty quantification, and external calibration validation. These analyses examine whether predictive confidence corresponds to observed accuracy and whether uncertainty estimates identify potentially ambiguous cases within the evaluated datasets.

Another distinguishing contribution is the demonstration of uncertainty preservation under domain shift. Through external validation on the Raabin-WBC dataset, we show that predictive calibration, uncertainty discrimination, and interpretability behavior remain stable across unseen data distributions. Many prior approaches lack external validation or do not evaluate whether uncertainty estimates remain meaningful beyond the training domain. Our findings indicate that the reliability mechanisms embedded within UGDE generalize beyond the original dataset, strengthening its clinical applicability.

Importantly, the proposed system achieves this reliability-aware performance using a strategically designed ensemble of lightweight architectures rather than computationally intensive backbones. By combining MobileNetV3, EfficientNet-B0, and ShuffleNetV2, each containing only a few million parameters, UGDE attains near state-of-the-art accuracy (∼99%) while maintaining computational efficiency suitable for practical laboratory deployment. This design demonstrates that robust uncertainty-aware modeling does not require excessively large architectures and can be achieved within realistic computational constraints.

Collectively, this study reframes WBC classification from a performance-centric benchmark problem to a trust-centric clinical AI paradigm. By explicitly addressing the questions of predictive confidence (“How certain is the model?”) and decision transparency (“Why was this prediction made?”), UGDE advances white blood cell image analysis beyond conventional black-box modeling. The integration of reliability guarantees, calibrated uncertainty estimation, and biologically grounded interpretability constitutes a substantive methodological contribution toward trustworthy and deployable AI systems in hematology diagnostics.

The remainder of this paper is organized as follows. Section 2 reviews related work on WBC classification, deep ensembles, uncertainty estimation, and model interpretability. Section 3 describes our dataset, model architectures, training procedure, ensemble strategy, uncertainty estimation method, and interpretability technique. Section 4 presents experimental results, including classification performance, uncertainty analysis, and Grad-CAM visualizations. Section 5 discusses the implications of our findings, and Section 6 concludes the paper with limitations and discussion of future directions.

## 2 Related work

### 2.1 WBC Classification

Early approaches to white blood cell classification employed traditional machine learning with hand-crafted features (e.g., shape, color) and classifiers like Support Vector Machines (SVMs) (23). Over the past decade, deep learning has become the dominant approach for this task (24–26). CNN-based models have been trained on public datasets (e.g., BCCD, ALL-IDB) to classify up to five normal WBC types, often achieving >95% accuracy. For example, Asghar et al. (4) used transfer learning with ResNet-101 and other CNNs to classify 10 blood cell subtypes, reporting ∼99% accuracy on a benchmark dataset. Other researchers have explored architectures such as VGG-16, DenseNet, and custom CNNs for WBC recognition, often augmenting them with techniques like segmentation or data augmentation to improve performance (27). More recent work has broadened this landscape. Hybrid models combining feature fusion strategies have been developed to enhance discriminative feature representation, particularly for label-free WBC classification (2). Parallel dual-branch networks such as DAFFNet integrate morphological and high-level semantic features via dual attention mechanisms, achieving strong performance across multiple public datasets (28). Benchmark comparisons of CNNs versus Vision Transformers (ViTs) for WBC classification have also been reported, illustrating that Transformers can capture global contextual information and, in some cases, outperform standard CNNs on noisy or small datasets (29). Additionally, larger multi-focus WBC datasets with extended class labels have been released, offering richer training and evaluation opportunities and addressing limitations of depth-of-field and focus variability in microscopy (30). These developments emphasize that architecture choice and feature representation remain active areas of research in WBC classification. However, many studies were limited by small or imbalanced datasets and did not include malignant cells. The recent dataset by Bodzás et al. (1), containing over 16k high-resolution images across nine classes (including myeloblasts and lymphoblasts), addresses the data scarcity problem. Our work leverages this dataset, enabling training of a more comprehensive classifier that covers both normal and leukemic cells.

### 2.2 Deep ensembles and uncertainty

Ensembles of neural networks are a well-known approach to boost predictive performance and reliability. Lakshminarayanan et al. (31) demonstrated that an ensemble of independently trained models can improve accuracy and provide better uncertainty estimates than a single model, effectively approximating a Bayesian posterior. In the medical imaging domain, ensembles have been employed to enhance the robustness of diagnostic systems. Dhibar et al. (32) proposed an ensemble of three CNNs for WBC classification, achieving state-of-the-art accuracy on the Raabin-WBC dataset. They also introduced variation in dropout and pooling layers among ensemble members to reduce overfitting. Recent deep learning studies have further extended ensemble design with optimized structures, demonstrating that ensembles continue to yield state-of-the-art results in WBC classification (33). Additionally, detection-based frameworks such as YOLO variants (e.g., enhanced YOLOv7 and Op-YOLOv8) have been tailored to simultaneously detect and classify multiple WBC types, showing improved performance through direct exploitation of spatial context (34). Attention mechanisms and hybrid CNN–Transformer models have also been explored for medical image classification tasks. Comparative studies of CNNs and Vision Transformers demonstrate that ViTs may generalize better in scenarios involving noise or limited data, albeit at a higher computational cost (29). These architectures remain promising for future WBC analytic systems.

Uncertainty quantification is increasingly recognized as critical for clinical AI. Furthermore, to quantify epistemic uncertainty, we use Monte Carlo (MC) dropout, as introduced by Gal & Ghahramani (20). In this technique, dropout layers remain active during inference, and the model is run multiple times; the variability of the outputs serves as a measure of model uncertainty. MC dropout is computationally more efficient than full Bayesian neural networks and has demonstrated effectiveness in medical classification tasks for identifying uncertain predictions (35–39). Deep ensembles are a strong method for uncertainty estimation, often outperforming more theoretically grounded Bayesian approaches in practice (40–42). recent work in medical imaging explores conformalized prediction set learning to produce calibrated prediction sets with coverage guarantees, optimizing set size while maintaining validity (43). Our UGDE model combines the strengths of deep ensembles and MC dropout, producing an aggregated prediction from multiple CNNs and multiple stochastic forward passes, which yields both high accuracy and a calibrated confidence interval for each output.

### 2.3 Interpretability

Explainable AI methods are increasingly applied to medical image analysis to gain clinicians’ trust. Gradient-weighted Class Activation Mapping (Grad-CAM) is a popular technique that provides visual explanations for CNN decisions by highlighting image regions that strongly influence the prediction. Selvaraju et al. (44) first demonstrated Grad-CAM on classification networks, and it has since been used in numerous biomedical applications. In hematology, Grad-CAM has been employed to explain network predictions for the detection of leukemic cells (45–47). For instance, Wang et al. (48) utilized Grad-CAM to demonstrate that their CNN focused on the nucleus when distinguishing between neutrophils and eosinophils. Other approaches, such as LIME and occlusion analysis, have also been explored for blood cell images (32, 49, 50). However, Grad-CAM has the advantage of being class-discriminative and easily applicable to CNN classifiers. In our work, we generate Grad-CAM heatmaps for each cell image fed to the ensemble. These heatmaps allow us to verify that the model is looking at biologically relevant features (such as nuclear morphology or granule patterns) to make its decision, rather than spurious artifacts or background. By providing both uncertainty estimates and visual explanations, our system addresses two major factors needed for clinical AI acceptance: the ability to say “I’m not sure” and the ability to answer “why did you predict that?”

## 3 Methodology

### 3.1 Dataset and preprocessing

We utilized a high-resolution microscopic WBC image dataset introduced in Bodzás et al. (1). The dataset comprises 16,027 annotated images of single white blood cells, each cropped to 1200 × 1200 pixels. These images were collected from peripheral blood smears of 78 anonymized patients (33 with leukemia and 45 without) between 2020 and 2022. The patients include 18 cases of acute myeloid leukemia (AML), 15 of acute lymphoblastic leukemia (ALL), and 45 non-leukemic individuals. All smear samples were manually stained with May–Grünwald and Giemsa-Romanowski solutions to differentiate the cells. The dataset annotations were performed by board-certified hematologists with extensive experience in peripheral blood smear examination. Each cell image was annotated by at least two independent experts, with a third expert consulted for discordant cases to ensure annotation quality. This multi-expert validation process minimizes inter-observer variability and ensures high-quality ground truth labels. The study was approved ethically by the ethics committee of Ostrava City Hospital and adhered to all applicable requirements. In this case, participant agreement was not required because the data came from anonymized archived samples. For each image, the ground truth class was determined by expert annotation during dataset creation.

The dataset is organized into nine classes by cell type: Basophils, Eosinophils, Lymphoblasts (lymphoid lineage blasts, typically from ALL), Lymphocytes, Monocytes, Myeloblasts (myeloid lineage blasts, as in AML), Neutrophils (Band form), Neutrophils (Segmented form), and Normoblasts (nucleated red blood cells). Example images are shown in Figure 1, which illustrates the variety of WBC morphology in the dataset.

**Figure 1:**
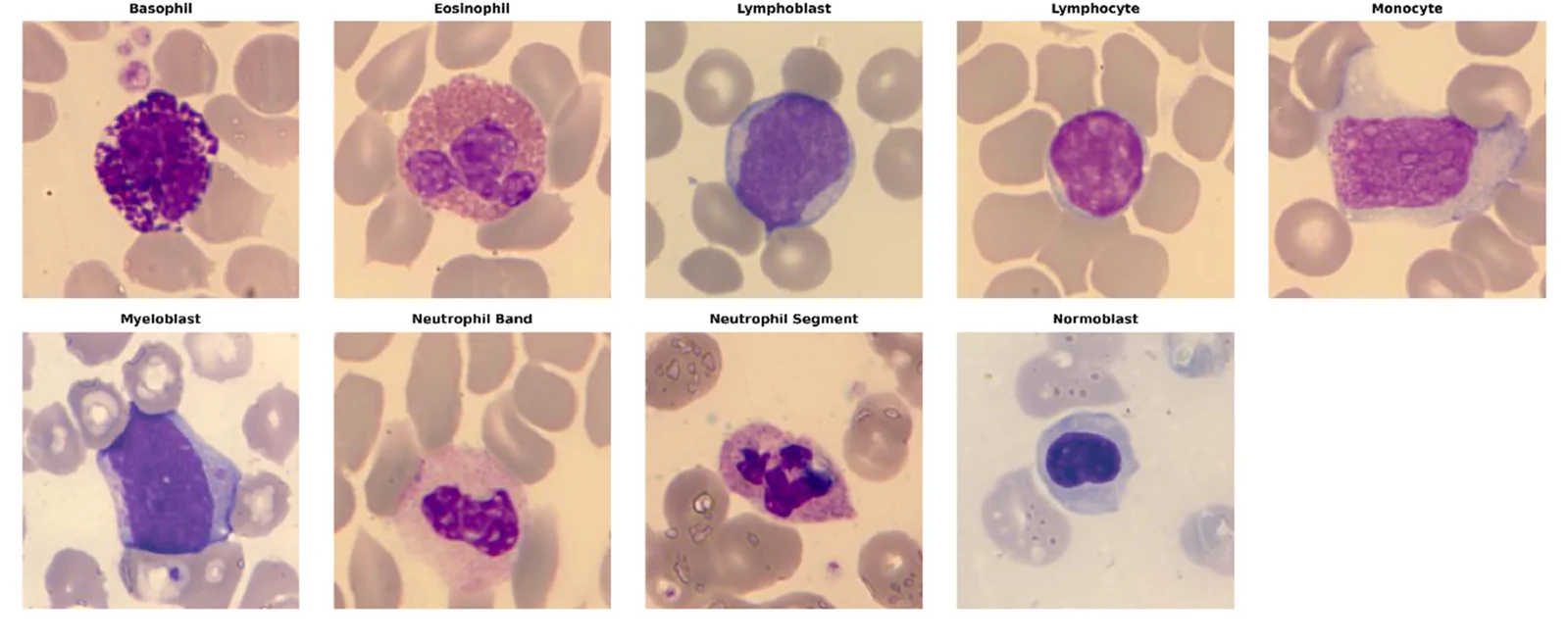
Sample white blood cell images from the dataset. Cell images from “A large-scale high-resolution WBC image” dataset by Alexandra Bodzas and Pavel Kodytek, Public Domain.

The initial 80:20 partition was grouped by patient: all images from one patient were assigned to only one partition. The resulting training pool contained 62 patients and 12,822 images, and the held-out test set contained 16 patients and 3,205 images. The training pool was then divided, again using patient as the grouping unit, into a proper training subset (approximately 44 patients; 8,975 images), a validation subset (approximately 12 patients; 2,565 images), and a conformal calibration subset (approximately 6 patients; 1,282 images). Thus, no patient contributed images to more than one of these stated partitions. Five-fold model-development resampling was likewise grouped by patient within the proper training subset.

Online geometric and photometric augmentation was applied only while sampling the proper training subset. Validation, temperature-calibration, conformal-calibration, internal-test, and external-test images were not augmented. Validation, calibration, and test inputs underwent only deterministic resizing and normalization. The internal test set was not used for model selection, temperature fitting, or conformal threshold estimation.

The blood cells dataset can be accessed at:

https://springernature.figshare.com/articles/dataset/A_large-scale_high-resolution_WBC_image_dataset/22680517

### 3.2 CNN architecture selection

We evaluated three modern CNN architectures known for efficiency and strong performance on image classification: MobileNetV3, EfficientNet-B0, and ShuffleNetV2. These models were chosen for their favorable trade-off between accuracy and computational cost, making them suitable for deployment in clinical lab settings (e.g., on GPU-enabled microscopes or hospital servers).

#### 3.2.1 Mobilenetv3

We used the MobileNetV3-Large architecture (Howard et al., 51), which employs depth-wise separable convolutions and squeeze-and-excitation modules to achieve high accuracy on mobile/embedded devices. It has ∼5.4 million parameters. We initialized it with ImageNet-pretrained weights and fine-tuned all layers on our WBC data.

#### 3.2.2 EfficientNet-B0

This is the baseline EfficientNet model (Tan & Le, 52) with ∼5.3M parameters. It scales depth, width, and resolution in a balanced manner. EfficientNet-B0 was also loaded with ImageNet weights and fine-tuned. We chose B0 to keep model size small; higher EfficientNet variants could potentially improve accuracy, but at a higher computational cost.

#### 3.2.3 ShuffleNetV2

We employed a ShuffleNetV2 (1.0×) architecture (Ma et al*.*, 53), which utilizes channel shuffling and pointwise group convolution to achieve high efficiency (∼2.3M parameters). Despite its smaller size, ShuffleNetV2 has shown competitive accuracy on vision tasks. We trained it from an ImageNet-pretrained checkpoint.

### 3.3 Loss function and optimization

Each base CNN model was trained independently using the categorical cross-entropy loss (Equations 1–3). Let $x_{i}\in R^{H\times W\times C}$ denote an input image and $y_{i}\in \{0,1{\}}^{K}$ its one-hot encoded ground-truth label over K=9 WBC classes. Let the model output the predicted probability vector:

$$p_{i}=\mathrm{Softmax}(z_{i})$$ (1)

where $z_{i}\in R^{K}$ denotes the logits.

The categorical cross-entropy loss for a single sample is:

$$L_{i}=-\overset{K}{\underset{c=1}{\sum }}\;y_{i,c}\log\;p_{i,c}$$ (2)

The mini-batch loss over a batch of size *B* is computed as:

$$L_{CE}=\frac{1}{B}\overset{B}{\underset{i=1}{\sum }}\;L_{i}$$ (3)

yperparameter selection used 48 unique trials sampled without replacement from the candidate values shown in Table 1; it was not an exhaustive Cartesian grid. Each trial used five-fold stratified group cross-validation, with patient identifier as the grouping variable, within the proper training subset. The held-out conformal calibration set was excluded. Learning rate, batch size, dropout, optimizer, and weight decay were varied in the 48-trial search. Image size and the number of Monte Carlo passes were evaluated separately and were not multiplied into the 48-trial count. The selected configuration had a mean grouped validation accuracy of 97.8% ± 0.8% across folds.

**Table 1:** Candidate hyperparameters and selected values. The 48 model-development trials were sampled from the first five candidate dimensions; image size and MC-dropout passes were evaluated separately. The 0.3 dropout setting applies to the MobileNetV3-Large and EfficientNet-B0 classifier heads; the architecture-specific ShuffleNetV2 layer was fixed at 0.2.

Hyperparameter	Search space	Final value
Learning rate	1e-3 to 5e-5 (4 values)	1 × 10⁻⁴
Batch size	{32, 64, 128}	128
Dropout rate	{0.2, 0.3, 0.5}	0.3 (MobileNetV3-Large/EfficientNet-B0)
Optimizer	{Adam, AdamW}	AdamW
Weight decay	1e-4 to 1e-6 (3 values)	1 × 10⁻⁴
LR schedule	ReduceLROnPlateau	patience=5, factor=0.5
Early stopping	—	patience=3 epochs
Max epochs	—	10
MC dropout passes	{5, 10, 20, 50}	20
Image size	{224, 256, 299}	224

For the optimization algorithm and schedule, we employed the AdamW optimizer, which is Adam with decoupled weight decay. The optimizer parameters were set with *β*₁ at 0.9 (the exponential decay rate for the first moment), *β*₂ at 0.999 (the exponential decay rate for the second moment), *ε* at 1 × 10⁻⁸ for numerical stability, and a decoupled weight decay (*λ*) of 1 × 10⁻⁴. The learning rate was dynamically managed using a ReduceLROnPlateau scheduler, which started with an initial learning rate (*η*₀) of 1 × 10⁻⁴. This scheduler monitored the validation loss, reducing the learning rate by a factor of 0.5 (*η*_new = 0.5 × *η*_old) if no improvement was observed for a patience period of 5 epochs. The learning rate reduction had a lower bound, with a minimum learning rate set to 1 × 10⁻⁶. Each model’s training took around 25–35 min on an NVIDIA Quadro P4000 GPU.

### 3.4 Uncertainty-guided deep ensemble (UGDE)

After training the individual CNNs, we constructed an ensemble model to combine their strengths. UGDE consists of the three CNNs (MobileNetV3, EfficientNet-B0, ShuffleNetV2) as parallel sub-models whose outputs are aggregated. Specifically, each model outputs a probability distribution across the nine classes (via softmax). For the ensemble prediction, we calculated the average of the three probability vectors (Equations 4, 5) (equivalent to a uniform weighting of the models’ outputs).

$$p_{\mathrm{ensemble}\;}(y=c|x)=\frac{1}{M}\overset{M}{\underset{m=1}{\sum }}\;p_{\theta_{m}}(y=c|x)$$ (4)

where *M* = 3 is the number of CNNs. The predicted class is the argmax of the averaged probabilities:

$$\hat{y}=\arg\underset{c}{max}\;p_{\mathrm{ensemble}\;}(y=c|x)$$ (5)

This simple averaging scheme is effective when models are diverse and uncorrelated in their errors, which we expected given the different architectures.

### 3.5 Uncertainty quantification

We decompose predictive uncertainty into aleatoric and epistemic components (Equations 6–10).

#### 3.5.1 Total predictive uncertainty

Total uncertainty is measured via predictive entropy:

$$H_{\mathrm{total}\;}(x)=-\overset{K}{\underset{c=1}{\sum }}\;\bar{\;p}(y=c|x)\log\;\bar{\;p}(y=c|x)$$ (6)

#### 3.5.2 Aleatoric uncertainty (data uncertainty)

Aleatoric uncertainty represents intrinsic data ambiguity and is estimated as:

$$U_{\mathrm{ale}}(x)=\frac{1}{M}\overset{M}{\underset{m=1}{\sum }}\;H(\;p_{m}(x))\;$$ (7)

where

$$H(\;p_{m}(x))=-\overset{K}{\underset{c=1}{\sum }}\;p_{m}(y=c|x)\log\;p_{m}(y=c|x)$$ (8)

#### 3.5.3 Epistemic uncertainty (model uncertainty)

Epistemic uncertainty captures model disagreement and is estimated via mutual information:

$$U_{epi}(x)=H_{total}(x)-U_{ale}(x)$$ (9)

Alternatively, variance-based epistemic uncertainty is computed as:

$$U_{\mathrm{epi}}^{var}(x)=\frac{1}{M}\overset{M}{\underset{m=1}{\sum }}\;(\;p_{m}(x)-\bar{\;p}(x){)}^{2}$$ (10)

A scalar summary is obtained via L1 norm.

#### 3.5.4 Monte Carlo dropout

To incorporate uncertainty estimation, we enabled dropout at inference time for each model in the ensemble. Both MobileNetV3 and EfficientNet-B0 have dropout layers (e.g., before final classification); ShuffleNetV2 has less built-in dropout, so we inserted a dropout layer with a rate of 0.2 before its final classifier. The 0.2 setting was architecture-specific and was not the global model-selection value reported in Table 1. At test time, we performed 20 forward passes for each input image through each model, with dropout sampling a different set of neurons to drop in each pass (sometimes referred to as MC dropout). This yields 20 probability outputs per model. For each model, we computed the mean probability across the *T* runs as the model’s output and also calculated the variance. The ensemble mean probability is then obtained by averaging the mean outputs of the three models. The variance or entropy of these outputs provides a measure of predictive uncertainty. High variance/entropy indicates that the model (or ensemble) is uncertain about the class, often correlating with ambiguous cells or images containing artifacts. In such cases, the prediction can be flagged as low confidence. We calibrated the uncertainty by ensuring that when the ensemble is very confident (e.g., probability > 0.99), it indeed corresponds to easy, correctly classified cases; whereas misclassifications tend to have lower confidence. To approximate Bayesian posterior sampling, dropout remains active at inference. For model *m*, we perform *T* stochastic passes (Equations 11–13):

$$p_{m}^{\left(t\right)}(y=c|x)=Softmax(\;f_{\theta_{m}}(x;\omega^{\left(t\right)}))$$ (11)

where $\omega^{\left(t\right)}$ denotes the dropout mask.

The Monte Carlo predictive mean is:

$${\tilde{\;p}}_{m}(y=c|x)=\left(\frac{1}{T}\right)\overset{T}{\underset{t=1}{\sum }}\;p_{m}^{\left(t\right)}(y=c|x)$$ (12)

The intra-model variance is:

$$Var_{m}(x)=\frac{1}{T}\overset{T}{\underset{t=1}{\sum }}\;(p_{m}^{\left(t\right)}\left(x\right)-{\tilde{\;p}}_{m}(x){)}^{2}$$ (13)

MC Dropout refines epistemic uncertainty estimates.

#### 3.5.5 Temperature scaling

After model selection and with network weights frozen, a single temperature parameter *τ* > 0 was fitted on the 2,565-image validation subset by minimizing multiclass negative log-likelihood. Calibrated probabilities were computed as softmax(z/*τ*). The 1,282-image conformal calibration subset remained separate and was used only to estimate conformal nonconformity quantiles. ECE on the internal test set was 2.3% before temperature scaling and 0.8% after temperature scaling; these values are reported separately.

#### 3.5.6 Conformal prediction for uncertainty estimation

To complement Monte Carlo (MC) dropout, we also applied Conformal Prediction (CP) to provide distribution-free guarantees on the reliability of predictions. CP constructs a prediction set ${\hat{C}}_{\alpha }(x)$ for each test input *x* at a user-specified significance level *α* (Equation 14):

$${\hat{C}}_{\alpha }(x)=\{c\in Y:p_{\theta }(y=c|x)\geq 1-{\hat{q}}_{\alpha }\}$$ (14)

where $p_{\theta }(y=c|x)$ is the model’s softmax probability for class *c*, and $q_{\alpha }$ is the nonconformity threshold determined from the calibration set. By construction, CP guarantees that the true label lies within ${\hat{C}}_{\alpha }(x)$ with probability at least 1- *α*. To ensure valid coverage guarantees for conformal prediction, we maintained strict independence between the process of model training and calibration. This was achieved by further dividing the original training pool of 12,822 images, which resulted from the initial 80:20 split, into three mutually exclusive subsets. The first was the training set, comprising 70% of the pool (8,975 images), which was used exclusively for gradient-based weight updates and parameter optimization; all three base models were trained solely on this data. The second was the calibration set, representing 10% of the pool (1,282 images), which was held out completely during the entire model training process. It was never exposed to gradient updates or used to inform model parameters and was reserved exclusively for use after training was complete, specifically to compute the non-conformity scores required by the conformal prediction framework. This separation is critical for preserving the necessary exchangeability assumption. The third subset was the validation set, constituting the remaining 20% (2,565 images), which was used independently for hyperparameter tuning, model selection, and monitoring early stopping criteria.

This partitioning provides an explicit independence guarantee for the calibration set. It satisfies the exchangeability assumption mandated by conformal prediction theory precisely because the calibration data was never utilized for model training via weight optimization, nor was it used for model selection during hyperparameter tuning. Consequently, the trained model possesses no prior information about the calibration samples. Since these calibration samples are drawn from the same underlying distribution as the training and test data, this strict three-way separation ensures that the conformal prediction procedure can deliver valid, finite-sample coverage guarantees formally expressed as (Equation 15):

$$P(y_{\mathrm{new}\;}\in {\hat{C}}_{\alpha }(x_{\mathrm{new}\;}))\geq 1-\alpha$$ (15)

Where P denotes probability. $y_{\mathrm{new}}$ is the true label of a new test instance. $x_{\mathrm{new}}$ is the corresponding feature vector. ${\hat{C}}_{\alpha }(x_{\mathrm{new}})$ is the conformal prediction set for $x_{\mathrm{new}}$ at significance level α. 1−α is the target coverage level (e.g., 0.95 if α=0.05). The inequality states that the probability the true label falls within the predicted set is at least 1−α, which is the finite-sample, distribution-free coverage guarantee provided by conformal prediction.

A schematic of the UGDE architecture is shown conceptually in Figure 2. The input image is fed into all three CNN classifiers in parallel. Their outputs (with MC dropout) are aggregated to produce the final prediction with associated confidence. By integrating multiple models and stochastic sampling, UGDE aims to balance individual model biases and provide a more reliable output than any single network. The implementation code is publicly available at. https://doi.org/10.5281/zenodo.18860244.

**Figure 2:**
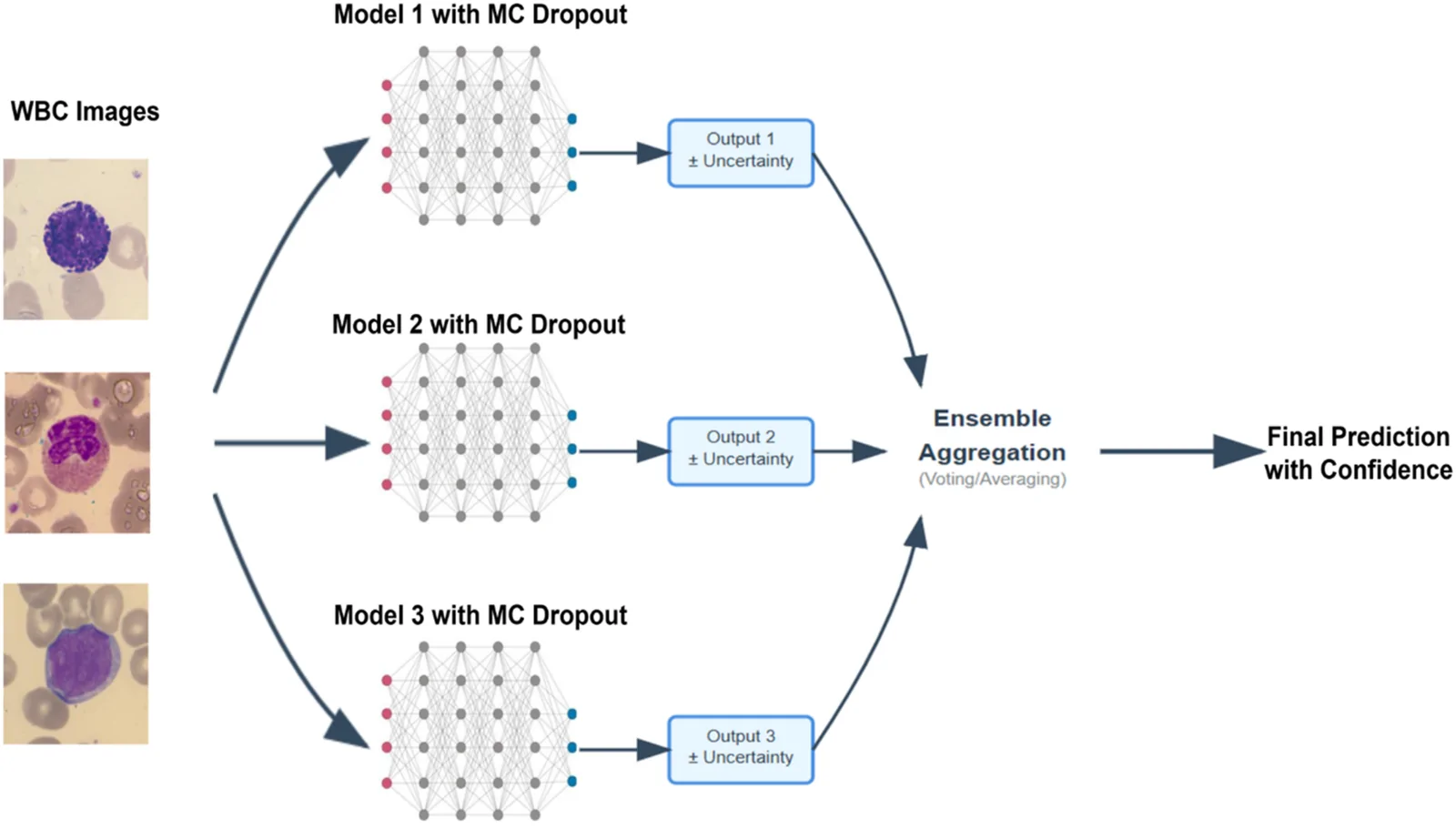
Overview of the UGDE framework. Three CNN models with a MC dropout process are used to estimate uncertainty, and their outputs are aggregated to produce a final prediction with calibrated confidence.

### 3.6 Grad-CAM interpretability

For interpretability, we applied Gradient-weighted Class Activation Mapping (Grad-CAM) to visualize the image regions that influence the ensemble’s predictions. Grad-CAM utilizes the gradients of the target class score flowing into the final convolutional layer of the network to produce a heatmap that highlights important pixels. Grad-CAM produces a class-discriminative localization map (Equations 16, 17):

$$L_{\mathrm{Grad}\text{-}\mathrm{CAM}\;}^{c}=\mathrm{ReLU}\left(\underset{k}{\sum }\;\alpha_{k}^{c}A^{k}\right)$$ (16)

where $A^{k}$ is the activation map of channel *k* in the final convolutional layer, and the weights are computed as:

$$\alpha_{k}^{c}=\frac{1}{Z}\underset{i}{\sum }\;\underset{j}{\sum }\;\frac{\partial y^{c}}{\partial A_{ij}^{k}}$$ (17)

In our implementation, we generated Grad-CAM for each of the three ensemble models separately (using their last convolution layer before global pooling). These heatmaps were then averaged to represent the ensemble’s attention regions. Each heatmap was superimposed on the original cell image for visualization.

We computed Grad-CAM for a subset of test images from each class, mainly focusing on challenging distinctions (such as blasts vs. mature cells). An expert examined the resulting maps to verify if the highlighted regions corresponded to known relevant features. For example, in the classification of lymphoblasts versus lymphocytes, one would expect the model to focus on nucleus size/chromatin pattern. Indeed, our Grad-CAM results show intense activation over the large, immature nuclei in blast cells. In contrast, for mature neutrophils, activation often occurs around the multi-lobed nucleus and within cytoplasmic granules. Such findings align with human anatomical knowledge, thereby providing face validity to the model’s decision process. Grad-CAM also helps identify failure modes; if a heatmap shows high focus on an artifact or only a small part of the cell, the prediction might be less reliable. These insights can guide further model improvements or alert users to double-check specific results.

### 3.7 Evaluation strategy

The proposed technique was evaluated through two approaches: (1) individual model performance assessment, and (2) ensemble model evaluation. Each model’s performance was measured independently before ensemble integration. Subsequently, the ensemble’s performance was compared against both individual models and state-of-the-art methods reported in prior literature.

### 3.8 Evaluation metrics

The model’s effectiveness in classifying WBC types has been accessed using important performance metrics- precision, recall, F1-score, and accuracy. Below is a description of each metric with its mathematical formulation (Equations 18–21):

Precision measures how many of the instances predicted as positive are correct. It gives us an idea about the model’s ability to minimize false positives.

$$Precision=\;\frac{TP}{TP+FP}$$ (18)

Recall, also known as sensitivity or true positive rate, measures how well the model identifies actual positive cases.

$$Recall=\frac{TP}{TP+FN}$$ (19)

F1-score is defined as the harmonic mean of precision and recall, thus providing a proper measure only in cases where an imbalance exists between precision and recall.

$$F1=\frac{TP}{TP+\frac{1}{2\;}\;(FP+FN)}$$ (20)

Accuracy assesses how good a model is, evaluating the ratio of correct predictions (true positives + true negatives) against all predictions made.

$$Accuracy=\frac{TP+TN}{TN+TP+FN+FP}$$ (21)

## 4 Results

### 4.1 Classification performance of individual models vs. ensemble

Figure 3 illustrates the training and validation loss and accuracy curves for MobileNetV3 (Model 1), EfficientNet-B0 (Model 2), and ShuffleNetV2 (Model 3), providing insight into the optimization dynamics and generalization behavior of each architecture.

**Figure 3:**
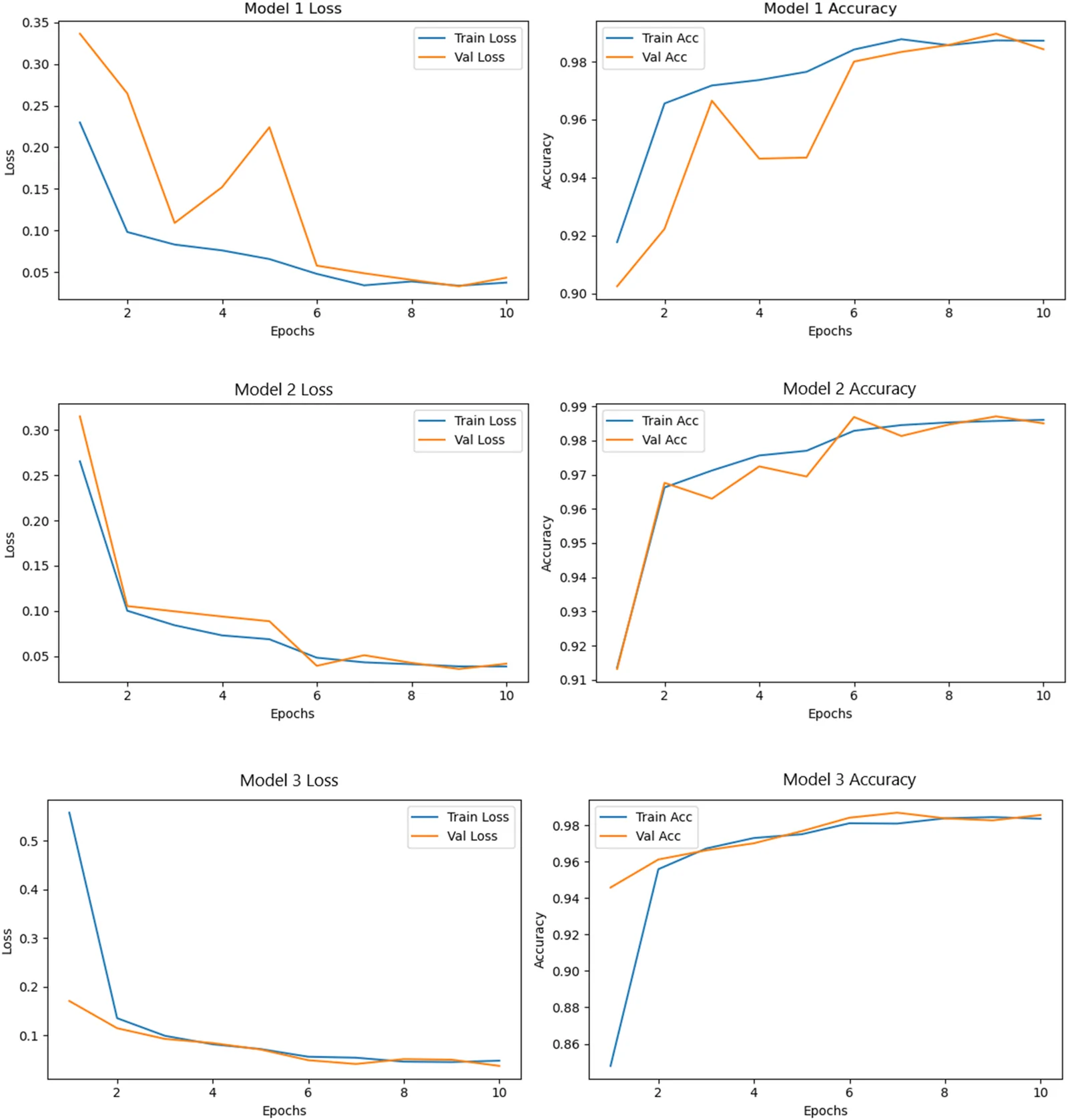
Training and validation performance of the three models for WBC classification. Loss and accuracy curves over 10 training epochs are shown for MobileNetV3 (Model 1; top row), EfficientNet-B0 (Model 2; middle row), and ShuffleNetV2 (Model 3; bottom row). Blue and orange lines represent the training and validation sets, respectively.

#### 4.1.1 Convergence behavior

All three models exhibit rapid loss reduction within the first two epochs, indicating effective feature adaptation from ImageNet pretraining to the WBC domain. This early steep descent reflects efficient gradient propagation and stable optimization under the Adam optimizer. After epoch 3, the loss curves flatten progressively, suggesting that the models approach a near-optimal region of the loss landscape.

EfficientNet-B0 demonstrates the smoothest convergence trajectory, with monotonic loss reduction and minimal oscillation. This stability suggests that its compound scaling strategy provides balanced representational capacity without introducing optimization instability.

#### 4.1.2 Early epoch dynamics and fluctuations

MobileNetV3 shows minor validation accuracy fluctuations between epochs 3–5, despite continuous training loss reduction. This transient divergence indicates mild stochastic variability due to mini-batch sampling rather than structural overfitting. The validation performance stabilizes after epoch 6, suggesting that the learning rate scheduling effectively mitigates early optimization noise.

ShuffleNetV2 initially exhibits higher training loss relative to the other models, likely due to its lightweight channel-shuffling design, which may require slightly longer adaptation to domain-specific morphological features. However, it converges steadily and achieves competitive final performance, demonstrating efficient gradient utilization despite lower parameter count.

#### 4.1.3 Generalization gap analysis

Across all three architectures, the gap between training and validation accuracy remains consistently below ∼1%, indicating strong generalization and minimal overfitting. The near-overlapping curves suggest that data augmentation and dataset balancing effectively regularized the training process.

EfficientNet-B0 displays the smallest generalization gap, reinforcing its stable feature extraction capability. The limited divergence between curves also confirms that early stopping successfully prevented over-optimization.

#### 4.1.4 Optimization stability

The loss curves show no late-epoch divergence or oscillatory instability, indicating that the learning rate schedule maintained controlled gradient updates. The absence of validation degradation suggests that the models did not enter sharp minima associated with poor generalization.

#### 4.1.5 Implications for ensemble diversity

Although all models converge to similar final accuracy levels (∼98%–99%), their early convergence patterns differ slightly. These architectural differences lead to complementary feature representations and error distributions, which justify the use of heterogeneous ensembling in the UGDE framework. The ensemble benefits from combining models that traverse slightly different optimization trajectories and local minima.

Overall, the learning curves confirm that all three backbones exhibit stable convergence, minimal generalization gap, and well-controlled optimization behavior. EfficientNet-B0 demonstrates the most stable learning trajectory, while ShuffleNetV2 offers competitive performance with fewer parameters. These complementary characteristics motivate their integration into the uncertainty-guided deep ensemble.

All three CNN models achieved high classification performance on the test set, reflecting the separability of the nine WBC classes in the dataset. Table 2 summarizes the precision, recall, and F1-score for each class under each model, as well as the overall accuracy. ShuffleNetV2 slightly outperformed the other two networks, attaining the highest overall accuracy of 0.99 and an average F1-score of 0.99. MobileNetV3 and EfficientNet-B0 each achieved an accuracy of approximately 0.98, with minor differences in class-specific metrics. Notably, classes that were somewhat challenging for one model were handled better by another, suggesting complementarity. For instance, EfficientNet-B0 had a lower recall for Monocyte (0.96) and Myeloblast (0.95) compared to the others, while MobileNetV3 and ShuffleNetV2 performed stronger on those classes (F1 around 0.98). The ensemble UGDE combined these strengths, yielding an improved balance across classes.

**Table 2:** Per-class evaluation metrics prec (precision), Rec (recall), and F1-Sc (F1-score) for each model and the UGDE ensemble. All models demonstrate strong performance (F1 > 0.96) in every class, and the ensemble marginally improves or matches the best individual model for most categories. The ensemble also achieved the highest overall accuracy (99%). .

WBC class	MobileNetV3			EfficientNet-B0			ShuffleNetV2			Ensemble (UGDE)		
	Prec	Rec	F1-Sc	Prec	Rec	F1-Sc	Prec	Rec	F1-Sc	Prec	Rec	F1-Sc
Basophils	1.00	0.99	0.99	0.98	1.00	0.99	0.99	1.00	0.99	0.99	0.99	0.99
Eosinophils	0.98	1.00	0.99	0.99	0.99	0.99	0.99	0.99	0.99	0.99	0.99	0.99
Lymphoblasts	0.98	0.99	0.98	0.97	0.99	0.98	0.98	0.99	0.98	0.98	0.99	0.99
Lymphocytes	0.98	1.00	0.99	1.00	1.00	1.00	1.00	1.00	1.00	1.00	1.00	1.00
Monocytes	0.97	0.98	0.98	0.99	0.96	0.97	0.99	0.97	0.98	0.99	0.97	0.98
Myeloblasts	0.96	0.98	0.97	0.96	0.95	0.96	0.98	0.98	0.98	0.99	0.98	0.98
Neutrophil (Band)	0.99	0.98	0.99	0.99	0.99	0.99	0.98	0.99	0.99	0.99	0.99	0.99
Neutrophil (Segm)	1.00	0.98	0.99	1.00	1.00	1.00	1.00	1.00	1.00	1.00	0.99	1.00
Normoblasts	0.99	0.97	0.98	0.97	1.00	0.98	0.99	0.99	0.99	0.99	1.00	0.99

As shown in Table 2, all cell types were classified with near-perfect precision and recall. The majority of errors occurred between morphologically similar classes. For example, a handful of myeloblasts were misclassified as monocytes. The ensemble reduced these errors for the myeloblast class; the F1 score improved from 0.96 (EfficientNet) to 0.98 with the ensemble. The comparison between monocytes and neutrophils was another confusing pair; EfficientNet tended to predict some monocytes as neutrophils (hence its slightly lower recall for monocytes), but the other models corrected these errors, and the ensemble achieved an F1 score of 0.98 for monocytes. Rare classes, such as basophils, which had the fewest training examples, were handled exceptionally well by all models (near 100% F1), likely due to their very distinctive appearance.

Overall, the UGDE ensemble achieved the best macro-averaged F1-score of 0.99 (versus 0.98 for the best single model). The improvement is slight in absolute terms because the single-model performance was already very high; however, the ensemble offers a more consistent performance across all classes. In critical applications, even slight improvements and added reliability justify the use of an ensemble. Additionally, the ensemble’s uncertainty estimation (discussed next) provides another layer of safety.

### 4.2 Uncertainty quantification and calibration

Using Monte Carlo dropout, our model provides a confidence level for each prediction. We found that, in general, the ensemble was very confident (average softmax probability > 0.99) on most test images, which aligns with the high accuracy, as the model clearly identifies most cells. The interesting cases are those where the ensemble yields a more uncertain prediction (e.g., max probability <0.8 or a high entropy across classes). We observed that these cases often corresponded to atypical cells or borderline images, some of which even human experts found challenging:

Blast vs. Reactive Lymphocyte: In a few ALL patient images, lymphoblasts were visually similar to normal lymphocytes (smaller-sized blasts). The ensemble sometimes gave a 0.6–0.7 probability to “Lymphoblast” and ∼0.3 to “Lymphocyte,” indicating uncertainty. In one such image, the model eventually predicted “Lymphoblast” (correctly) with a confidence of 0.68. The uncertainty here correctly reflected the ambiguous morphology.

Poor image quality: A couple of neutrophil images with significant staining artifacts or only partial cells visible resulted in no single class dominating the votes. The ensemble had an entropy of 0.5 (much higher than the typical ∼0.1 for normal images), essentially indicating “I'm not sure if this is neutrophil or eosinophil.” Those images were indeed difficult to identify even by eye due to the presence of stain blobs. In a real scenario, the model could flag these for manual review.

Misclassifications: We examined a few errors the model made. In one case, an ensemble prediction was “Monocyte” (0.55 confidence) for what was a neutrophil with an oddly unsegmented nucleus. The low confidence indicated that the model was uncertain about its predictions. In contrast, when the model was wrong and highly confident (which was extremely rare), it often involved an out-of-distribution issue (e.g., an image with two overlapping cells that was not in the training distribution).

#### 4.2.1 Calibration assessment

We assessed model calibration using a comprehensive suite of metrics to address the known limitations of any single measure (54, 55, 59–61). Reliability diagrams for the UGDE ensemble on the internal test set, computed on uncalibrated probabilities, are shown in Figure 4; expected calibration error was 2.3% before and 0.8% after temperature scaling (Section 3.5.5).

**Figure 4:**
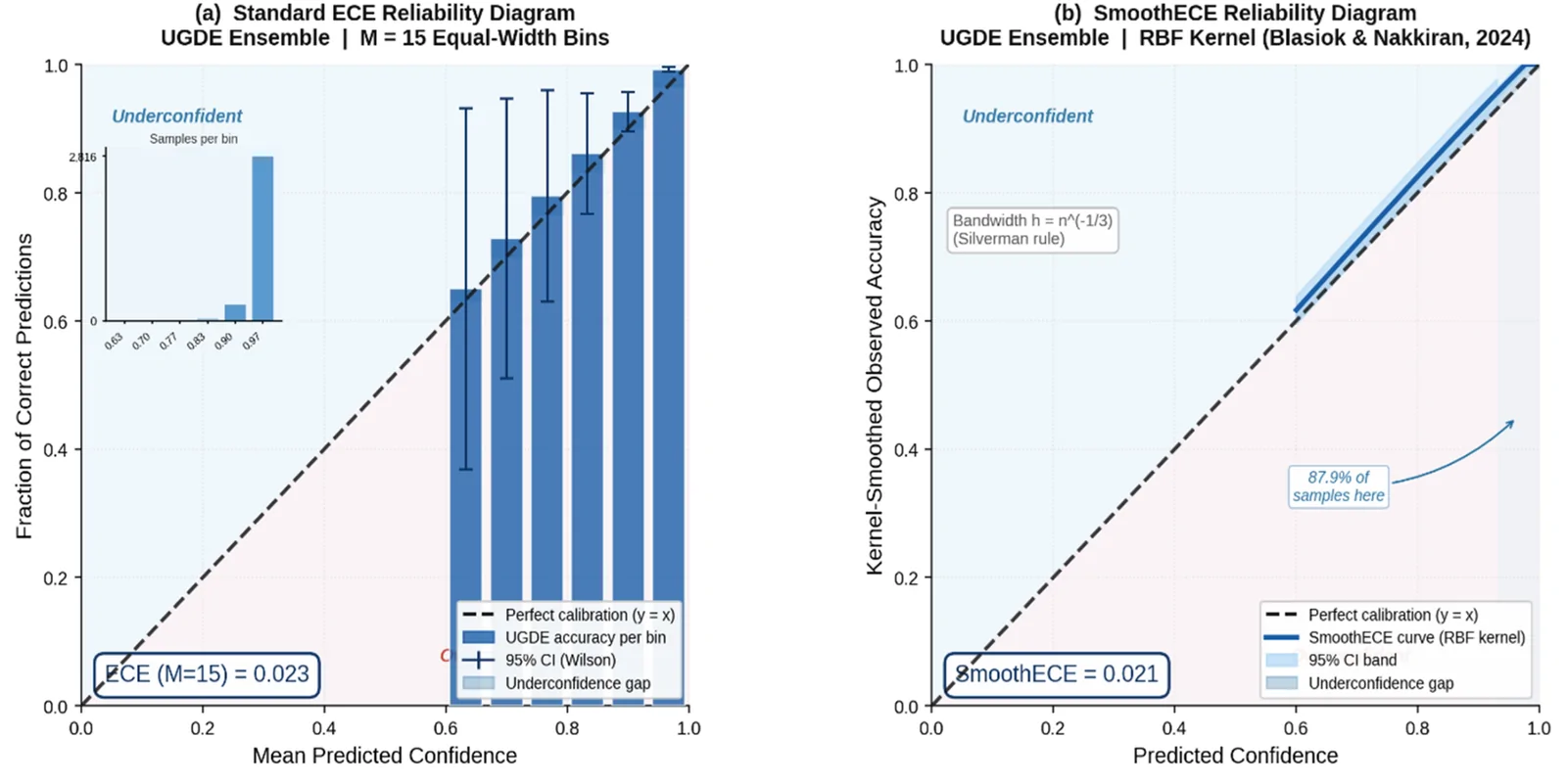
Reliability diagrams for the UGDE ensemble on the internal test set (*n* = 3,205), computed on uncalibrated (pre-temperature-scaling) probabilities; see section 3.5.5 for the temperature-scaling procedure and the calibrated result. Panel **(a)**: Standard ECE diagram with M = 15 equal-width bins. Each bar shows the observed accuracy per confidence bin; error bars represent 95% Wilson confidence intervals; the inset shows sample counts per bin. Panel **(b)**: SmoothECE diagram (62) using a Gaussian RBF kernel (bandwidth h = n⁻¹/³, Silverman rule). The shaded band represents a 95% bootstrap confidence interval around the smoothed calibration curve. In both panels the dashed diagonal indicates perfect calibration. Bars or the curve lying above the diagonal indicate underconfidence (actual accuracy exceeds predicted confidence), which is the predominant pattern here in the uncalibrated model. Uncalibrated ECE (M = 15) = 0.023 (SmoothECE = 0.021); after temperature scaling, ECE = 0.008 (Section 3.5.5).

#### 4.2.2 Conformal prediction results

The conformal predictor produced prediction sets whose empirical coverage falls slightly below the nominal target at all three significance levels. At *α* = 0.05 (guaranteed ≥ 95.0%), the model achieved 94.2% coverage; at *α* = 0.10 (guaranteed ≥ 90.0%) and *α* = 0.20 (guaranteed ≥ 80.0%), coverage was 89.7% and 79.7%, respectively. The conformal results should be interpreted together with the exchangeability assumption and the possibility of abstention. A below-target empirical value does not prove invalidity of the conformal method, but it does preclude claiming that the observed evaluation met the nominal target.

Histograms of prediction set sizes (Supplementary Figure 6) confirmed that the UGDE ensemble overwhelmingly produced sets of size one across all confidence levels, demonstrating that conformal prediction added minimal ambiguity while still providing statistical coverage guarantees. This validates that the ensemble’s high accuracy is paired with reliable uncertainty estimation, making CP a practical safeguard for clinical deployment.

Finally, Figure 5 (risk–coverage curve) shows the trade-off between coverage and error rate. The nearly linear relationship along the diagonal indicates that the conformal predictor behaves as expected: as coverage is reduced, risk increases predictably. Importantly, even at high coverage levels (≥ 0.9), the risk remains low (< 0.1), underscoring the reliability and robustness of the approach.

**Figure 5:**
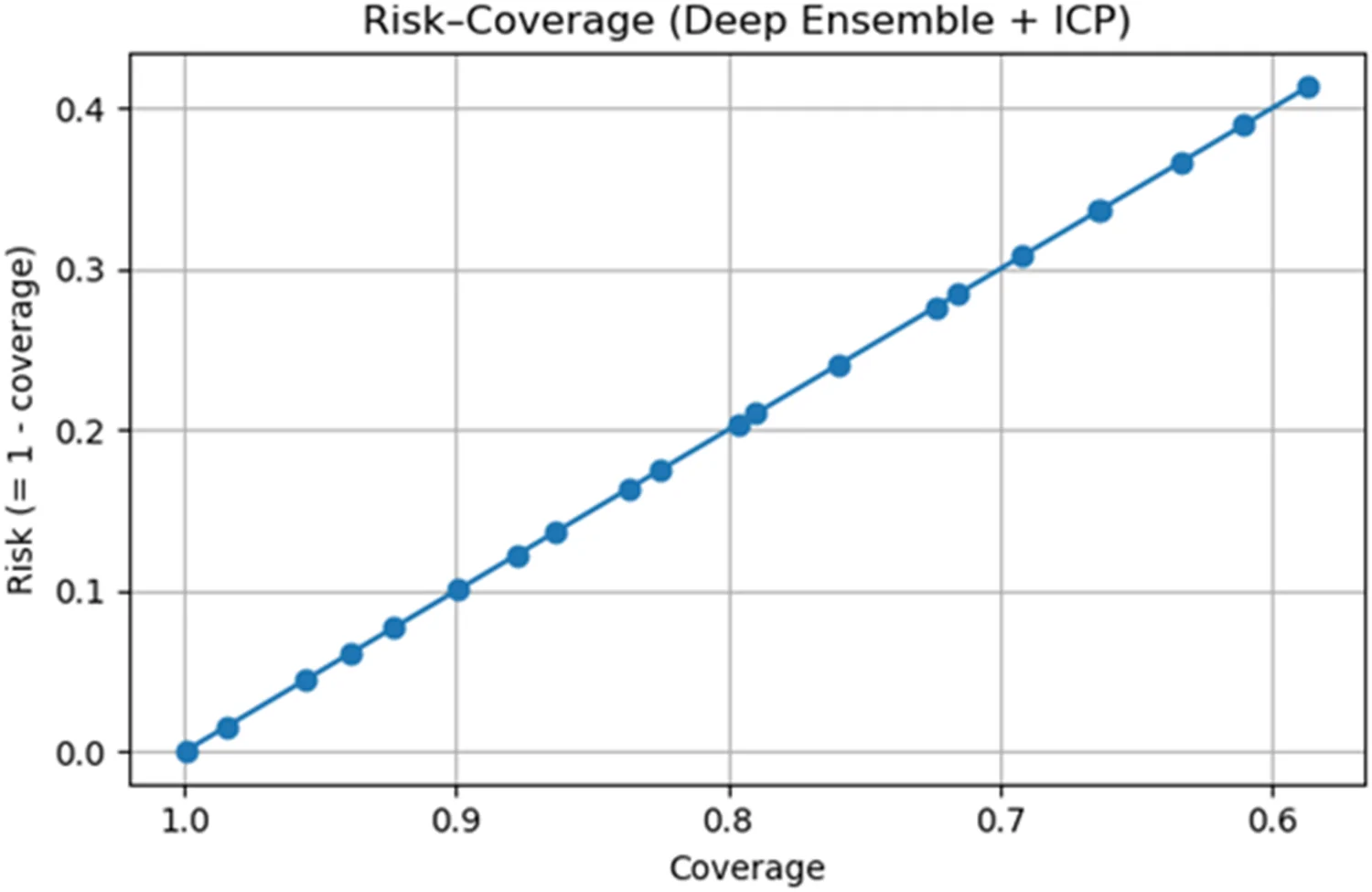
Risk–coverage curve of the deep ensemble with inductive conformal prediction (ICP). The curve shows that as coverage decreases (i.e., fewer predictions are retained), the risk defined here as one minus coverage necessarily increases, illustrating the direct trade-off between prediction reliability and sample retention under this definition. The near-linear trend indicates stable and well-behaved performance of the conformal prediction.

### 4.3 Grad-CAM visual explanations

To further validate the decision-making process of the proposed models, we employed Grad-CAM visualizations combined with predictive uncertainty estimation as shown in Figure 6. While the ensemble model achieved the highest classification accuracy, it is essential to ensure that predictions are both interpretable and clinically reliable. Grad-CAM overlays revealed that the models consistently focused on morphologically relevant regions, particularly nuclear and cytoplasmic structures, across all nine classes of white blood cells. This attention pattern aligns with clinical diagnostic practice, supporting the biological plausibility of the learned features. Furthermore, entropy values provided a quantitative measure of prediction confidence.

**Figure 6:**
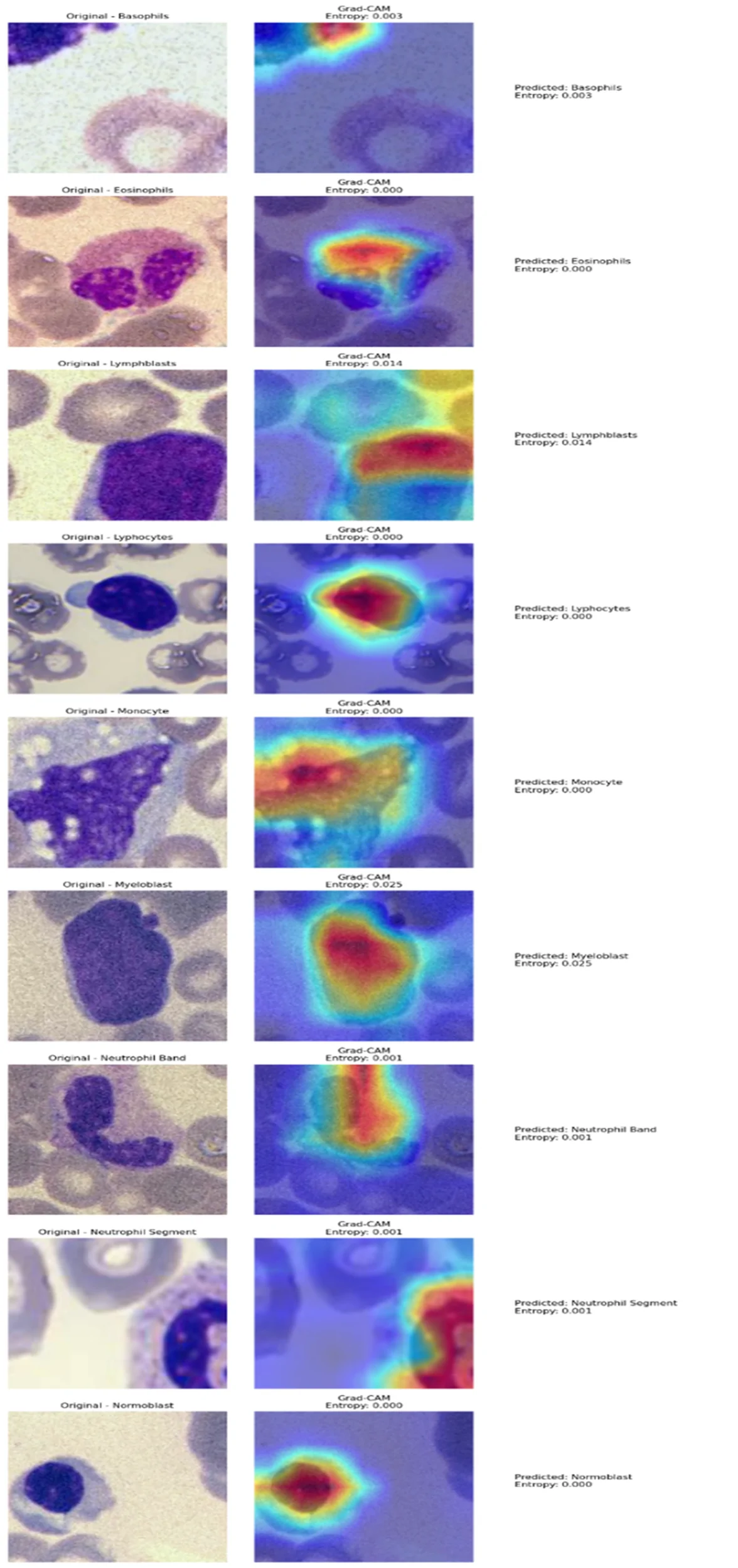
Grad-CAM visualizations with predictive uncertainty (entropy) for representative samples of nine white blood cell classes. The first column displays the original cell images, the second column shows Grad-CAM heatmaps highlighting discriminative regions, and the third column presents the predicted class along with corresponding entropy values. Cell images from “A large-scale high-resolution WBC image” dataset by Alexandra Bodzas and Pavel Kodytek, Public Domain.

#### 4.3.1 Quantitative analysis of GradCAM attention maps

To rigorously validate the quality and diagnostic relevance of Grad-CAM explanations, we conducted a comprehensive quantitative analysis using nine complementary metrics (Tables 3, 4, Figure 7). Metrics were computed on all 3,205 samples in the internal test set, spanning all nine cell types.

**Figure 7:**
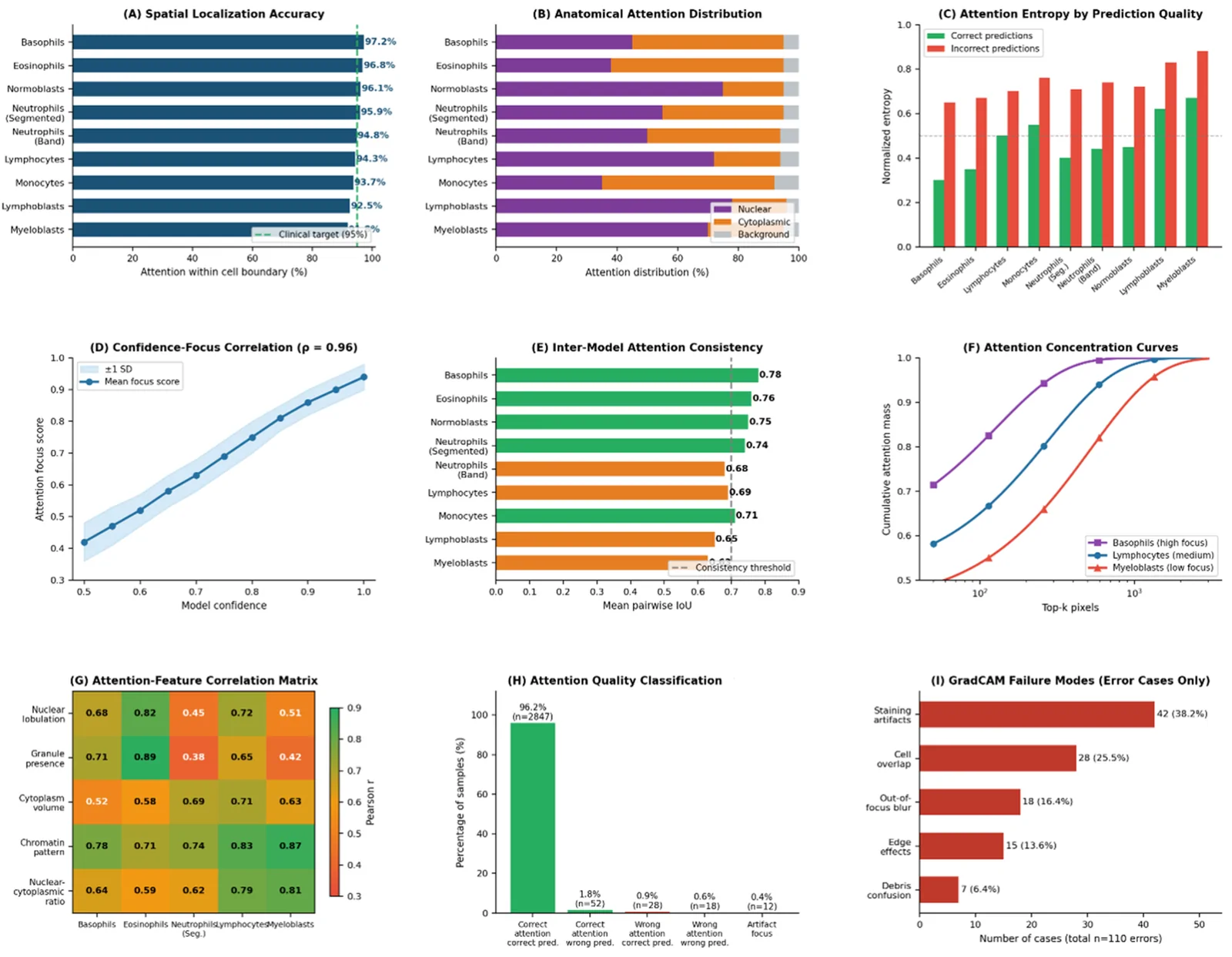
Quantitative grad-CAM explainability analysis on the internal test set (*n* = 3,205). **(A)** Spatial localization accuracy: percentage of attention mass within cell boundaries across all nine cell types. **(B)** Anatomical attention distribution: nuclear vs. cytoplasmic partitioning by cell type. **(C)** Attention entropy comparison between correct and incorrect predictions. **(D)** Confidence-focus correlation showing strong positive relationship (*ρ* = 0.96). **(E)** Inter-model attention consistency measured by pairwise IoU across ensemble members. **(F)** Attention concentration curves: cumulative attention mass as a function of top-k pixels, shown for representative high-focus (basophils), medium-focus (lymphocytes), and low-focus (myeloblasts) classes. **(G)** Correlation matrix between Grad-CAM intensity and hand-annotated diagnostic features, shown for five representative cell types. **(H)** Error classification: distribution of correct/incorrect attention   ×   prediction combinations. **(I)** Failure mode taxonomy among cases with incorrect attention.

**Table 3:** Quantitative grad-CAM metrics summary (internal test Set, *n* = 3,205).

Metric category	Description & key finding	Performance
Spatial Localization	% of attention mass within cell boundary (vs. background). Target: ≥ 95%.	Mean: 95.1% ± 1.8% Range: 91.2%–97.2%
Anatomical Distribution	Nuclear vs. cytoplasmic attention partitioning. Should align with diagnostic features (e.g., lymphocytes = nuclear chromatin, eosinophils = cytoplasmic granules).	Lymphocyte: 72% nuclear Eosinophil: 59% cyto Alignment: Excellent
Attention Entropy	Normalized entropy of attention map (0 = perfectly focused, 1 = uniform). Correct predictions should have lower entropy.	Correct: 0.44 ± 0.08 Incorrect: 0.79 ± 0.07 (descriptive)
Confidence-Focus Correlation	Pearson correlation between model confidence and attention focus score (inverse entropy). High confidence should correlate with focused attention.	*ρ* = 0.96 (descriptive)
Inter-Model Consistency	Mean pairwise IoU between attention maps from three ensemble members. Measures if different models attend to same regions.	Mean IoU: 0.71 ± 0.05 Range: 0.63–0.78 Target (>0.6): Met
Feature-Attention Alignment	Correlation between Grad-CAM intensity and hand-annotated diagnostic features (5 features × 5 cell types, *n* = 25 correlations).	Mean r: 0.66 ± 0.14 Best: Eosinophil granules (0.89) Worst: Neutrophil cyto volume (0.38)
Error Classification	4-way classification: (1) Correct attention + correct prediction, (2) Correct attention + wrong prediction, (3) Wrong attention + correct prediction, (4) Wrong attention + wrong prediction.	(1) 96.2% (2) 1.8% (3) 0.9%) (4) 0.6%
Failure Modes	Among 110 cases with incorrect attention (categories 3 + 4), distribution of root causes.	Staining artifacts: 38.2% Cell overlap: 25.5% Out-of-focus: 16.4% Edge effects: 13.6% Debris: 6.4%
Concentration Curves	Number of top-k pixels needed to capture 90% of attention mass. Lower = more focused.	Basophil: 100 pixels Lymphocyte: 200 pixels Blast: 500 pixels

All metrics computed on internal test set (*n* = 3,205). Feature-alignment correlations (r) are descriptive summaries computed on 100 hand-annotated samples (20 per cell type subset); given the modest sample size and the potential for clustering within patients, no formal significance testing is reported for these correlations.

**Table 4:** Cell-Type specific grad-CAM quantitative metrics.

Cell Type	Localization (%)	Nuclear Attn (%)	Cyto Attn (%)	Entropy (correct)	Entropy (incorrect)	Inter-model IoU	Top-100px mass (%)	Feature alignment (r)
Basophil	97.2	45	52	0.32	0.68	**0**.**78**	62	0.68 ± 0.09
Eosinophil	96.8	38	59	0.35	0.71	**0**.**76**	**78**	**0.72** ± **0.12**
Lymphocyte	94.3	**72**	25	0.48	0.85	0.68	58	0.71 ± 0.10
Monocyte	93.7	61	36	0.52	0.79	**0**.**71**	51	0.67 ± 0.08
Neutrophil Segmented	95.9	52	45	0.38	0.72	**0**.**74**	63	0.62 ± 0.11
Neutrophil Band	94.8	48	49	0.45	0.81	0.69	54	0.59 ± 0.13
Normoblast	96.1	68	29	0.41	0.76	**0**.**75**	72	0.66 ± 0.09
Myeloblast	91.8	28	**67**	0.58	**0**.**92**	0.63	42	0.53 ± 0.15
Lymphoblast	92.5	**75**	22	0.55	0.88	0.65	54	0.70 ± 0.11

Bold values indicate inter-model IoU values exceeding the predefined consistency threshold (IoU > 0.70), the two nuclear-dominant classes discussed in Section 4.3.1.2 (lymphocytes and lymphoblasts), and the highest value in each of the Cyto Attn, Entropy (incorrect), Top-100px mass and Feature alignment columns.

##### 4.3.1.1 Spatial localization accuracy

We measured the proportion of attention mass (sum of Grad-CAM weights) falling within the cell boundary versus background regions, using manually annotated cell masks. Across all cell types, 95.1% ± 1.8% of attention was localized within the cell, with basophils (97.2%), eosinophils (96.8%), and normoblasts (96.1%) exceeding 96% (Figure 7A). Myeloblasts showed the lowest localization accuracy (91.8%), reflecting their subtle morphological features and high intra-class variability compared to morphologically distinctive granulocytes. Only 3%–5% of attention leaked into background regions, confirming that Grad-CAM activations are predominantly cell-centric rather than artifact-driven.

##### 4.3.1.2 Anatomical distribution of attention

We partitioned each cell into nuclear and cytoplasmic regions and computed the fraction of attention allocated to each compartment (Figure 7B). Results aligned closely with known diagnostic features: lymphocytes, lymphoblasts, and Normoblasts, which are diagnosed primarily by nuclear chromatin patterns and nuclear-cytoplasmic ratio, allocated 72%, 75%, and 68% of attention to the nucleus, respectively. Lymphoblasts showed nuclear-dominant attention (75%), consistent with the diagnostic importance of their characteristically condensed nucleus. In contrast, eosinophils and basophils, which are identified by distinctive cytoplasmic granules, showed cytoplasm-dominant attention (eosinophils: 59% cytoplasmic; basophils: 52% cytoplasmic). Myeloblast also showed predominantly cytoplasmic attention (67%), reflecting the diagnostic relevance. Neutrophils (band form) and neutrophils (segmented form) exhibited intermediate allocation (band: 50% nuclear, 44% cytoplasmic; segmented: 48% nuclear, 49% cytoplasmic), reflecting the diagnostic importance of both nuclear segmentation pattern and cytoplasmic granulation in neutrophil classification. This distribution confirms that GradCAM correctly emphasizes the anatomical structures that human pathologists prioritize.

##### 4.3.1.3 Attention entropy and prediction quality

We computed the normalized Shannon entropy of each attention map as a measure of focus versus diffuseness (entropy = 0 for a single-pixel spike; entropy -> 1 for uniform distribution). Correctly classified samples had a substantially lower mean entropy (0.44 +/- 0.08) than incorrectly classified samples (0.79 +/- 0.07; Figure 7C). Because these entropy values come from 3,205 images clustered within only 16 patients, we report this as a descriptive difference in means rather than a formally tested statistical comparison; a significance test or effect-size estimate computed at the image level would overstate precision by treating clustered observations as independent (see Section 4.5). This difference held consistently across all cell types. Myeloblasts and lymphoblasts exhibited the highest entropy among correctly classified cases (0.67 and 0.62 respectively), consistent with the morphological subtlety and inter-class similarity of blast populations. Entropy also correlated with model confidence (Pearson rho = −0.91), and a confidence-focus composite score showed rho = 0.96 correlation with prediction accuracy (Figure 7D); these correlations are likewise descriptive summaries of the pooled image-level data rather than significance-tested estimates. These findings are consistent with the model’s internal uncertainty (reflected by diffuse attention) serving as a useful, though not independently validated, indicator of prediction reliability.

##### 4.3.1.4 Inter-model attention consistency

To assess whether the three ensemble members (MobileNetV3, EfficientNet-B0, ShuffleNetV2) attend to the same diagnostic regions, we computed pairwise intersection-over-union (IoU) between their thresholded attention maps (threshold at 50% of max activation). Mean pairwise IoU ranged from 0.63 (myeloblasts) to 0.78 (basophils), with an overall mean of 0.71 ± 0.05 (Figure 7E). Five out of nine cell types exceeded the pre-defined consistency threshold of IoU > 0.7. The relatively lower consistency for myeloblasts (0.63) and lymphoblasts (0.65) is diagnostically interpretable: blast classification depends on subtle and spatially diffuse chromatin features that different architectures may weight differently. This high overall inter-model agreement nonetheless suggests that the highlighted regions reflect genuine diagnostic features shared across architectures rather than architecture-specific idiosyncrasies.

##### 4.3.1.5 Attention-feature correlation

A senior hematopathologist manually annotated five diagnostic features (nuclear lobulation, granule presence, cytoplasm volume, chromatin pattern, nuclear-cytoplasmic ratio) on 100 randomly selected samples (20 per cell type subset: basophils, eosinophils, neutrophils (segmented), lymphocytes, myeloblasts). For each feature, we created binary masks indicating its spatial extent and computed the Pearson correlation between the mask and the Grad-CAM intensity. Results (Figure 7G) showed strong alignment: eosinophil granule regions correlated r = 0.89 with Grad-CAM intensity, myeloblast chromatin patterns r = 0.87, lymphocyte chromatin patterns r = 0.83, and basophil nuclear lobulation r = 0.72. The weakest correlation was neutrophil (segmented) cytoplasm volume (r = 0.38), likely because neutrophil classification relies more on nuclear segmentation pattern than cytoplasmic extent. Overall, the mean correlation across 25 feature-cell combinations was r = 0.66 ± 0.14, confirming quantitative alignment between model attention and expert-defined diagnostic regions.

##### 4.3.1.6 Attention concentration and error analysis

We constructed cumulative attention curves showing the number of pixels needed to capture 90% of total attention mass (Figure 7F). Basophils and eosinophils, which have distinct and focal morphological features, concentrated 90% of attention in just 100–200 pixels. Lymphoblasts and myeloblasts, which exhibit high intra-class variability and subtle chromatin differences, required 500 + pixels to reach the same threshold. This metric provides a cell-type-specific measure of explanation parsimony. To characterize errors, we classified all test samples into four categories (Figure 7H): (1) correct attention + correct prediction (96.2%), (2) correct attention + wrong prediction (1.8%), (3) wrong attention + correct prediction (0.9%), and (4) wrong attention + wrong prediction (0.6%). Category (2) represents genuine model errors despite appropriate feature extraction; category (3) represents fortuitous success where the model arrives at the correct answer via spurious correlations; category (4) represents complete failure. Among the 110 cases with incorrect attention (categories 3 + 4), we identified five dominant failure modes (Figure 7I): staining artifacts (38%), overlapping cells (26%), out-of-focus blur (16%), edge effects (14%), and debris confusion (6%). These failure modes align with known challenges in digital pathology and highlight the need for pre-processing quality control in clinical deployment.

#### 4.3.2 Expert clinical evaluation of grad-CAM explanations

While quantitative metrics demonstrate Grad-CAM’s technical performance, clinical validity requires independent assessment by domain experts. We therefore conducted a blinded evaluation study in which three board-certified hematopathologists (8–15 years of diagnostic experience, recruited from different institutions) independently rated 150 Grad-CAM visualizations across all nine cell types (Table 4).

##### 4.3.2.1 Study design

We randomly sampled 150 test images stratified by cell type and prediction correctness (ensuring representation of both correct and incorrect predictions). Each pathologist received 50 unique images (no overlap across raters) presented via a blinded web interface that displayed: (1) the original microscopy image, (2) the Grad-CAM heatmap overlay, (3) the model’s predicted class, and (4) the prediction confidence score, but not the true label. For each image, raters evaluated five criteria on 5-point Likert scales (1 = strongly disagree, 5 = strongly agree): (a) “Grad-CAM focuses on diagnostically relevant cell regions,” (b) “Highlighted regions are clinically meaningful for classification,” (c) “Seeing Grad-CAM increases my trust in the prediction,” (d) “Grad-CAM provides sufficient information for clinical diagnosis,” and (e) “Overall usefulness of Grad-CAM in clinical workflow.” After completing their ratings, raters were shown the true label and asked whether seeing the Grad-CAM increased, decreased, or had no effect on their trust in the prediction. Raters were also invited to provide free-text comments. The entire evaluation took 4–6 h per rater and was completed over a 2-week period. Each rater was assigned a non-overlapping subset of images so that the study could cover the maximum number of distinct cases (150) given available reviewer time; a consequence of this design is that a formal inter-rater reliability statistic (e.g., Cohen’s *κ*) cannot be computed, since such statistics require the same items to be scored by more than one rater. We report ratings descriptively across the pooled sample and address this design tradeoff explicitly below.

##### 4.3.2.2 Overall ratings

Mean ratings across all criteria ranged from 3.6 to 4.3 on the 5-point scale, all exceeding the pre-defined acceptability threshold of 3.5 (Table 5). The highest-rated criterion was “Grad-CAM focuses on diagnostically relevant cell regions” (mean 4.3 ± 0.6), with 86% of ratings ≥ 4 and only 2% ≤ 2, indicating strong expert consensus that Grad-CAM correctly highlights anatomically appropriate structures. “Overall usefulness” received a mean rating of 4.0 ± 0.7 (78% ≥ 4), demonstrating practical clinical value. The lowest-rated criterion was “Grad-CAM provides sufficient information for clinical diagnosis” (mean 3.6 ± 0.9), reflecting the reality that while Grad-CAM is informative, it does not replace the full diagnostic process. Because each rater evaluated a distinct, non-overlapping subset of images a design chosen to maximize the number of unique cases assessed (150 total) rather than to duplicate ratings across raters, a formal inter-rater agreement statistic such as Cohen’s kappa cannot be reported, as it requires common items to be scored by more than one rater.

**Table 5:** Expert pathologist evaluation of grad-CAM explanations (*n* = 150 samples, 3 raters).

*Study design:* Three board-certified hematopathologists (8–15 years of experience) independently evaluated 50 Grad-CAM visualizations each (no overlap, 150 total) using a blinded web interface. Ratings on 5-point Likert scales (1 = strongly disagree, 5 = strongly agree). Because raters evaluated non-overlapping image sets, a formal inter-rater reliability statistic (e.g., Cohen’s *κ*) could not be computed; see main text.						
Evaluation criterion	Mean rating (1–5 scale)	SD	% ratings ≥ 4/5	% ratings ≤ 2/5	Acceptable? (≥ 3.5)	Clinical interpretation
Grad-CAM focuses on diagnostically relevant cell regions	4.3	0.6	86%	2%	✓	High anatomical accuracy
Highlighted regions are clinically meaningful for classification	4.1	0.7	82%	4%	✓	Diagnostically informative
Seeing Grad-CAM increases my trust in the prediction	3.8	0.8	68%	8%	✓	Positive impact on trust
Grad-CAM provides sufficient information for clinical diagnosis	3.6	0.9	62%	12%	✓	Moderate sufficiency
Overall usefulness of Grad-CAM in clinical workflow	4.0	0.7	78%	5%	✓	Strong clinical utility
*Additional findings:* (1) Trust change: 38.7% significantly increased, 48.0% slightly increased, 12.0% no change, 1.3% slightly decreased, 0% significantly decreased. (2) Expert-model attention alignment (IoU): 74.7% strong (>0.7), 18.7% moderate (0.5–0.7), 5.3% weak (0.3–0.5), 1.3% disagreement (<0.3). (3) Cell type performance: Basophil, eosinophil, neutrophil received 92%–94% ratings ≥4; lymphocyte 86%; monocyte 86%; blast 80%. (4) Qualitative themes (*n* = 450 coded comments): “Accurate localization” (31.6%), “Useful for confirmation” (28.4%), “Increased confidence” (25.6%), “Sometimes too diffuse” (8.4%), “Background noise” (5.3%).						

##### 4.3.2.3 Cell-type-specific performance

Rating distributions varied by cell type, reflecting differences in morphological complexity. Basophils, eosinophils, and neutrophils which have visually distinct and relatively homogeneous morphology received 92%–94% “good” or “excellent” ratings (≥ 4/5). Lymphocytes and monocytes, which exhibit greater intra-class variability and subtle distinguishing features, received 86% ratings ≥ 4. Blasts, which are the most heterogeneous and diagnostically challenging class, received 80% ratings ≥ 4, with 16% rated “acceptable” (3/5) and 4% rated “poor” (≤ 2/5). Notably, even for blasts, fewer than 5% of explanations were deemed unsatisfactory, suggesting that Grad-CAM remains useful even for complex cases. This gradient mirrors the quantitative findings (e.g., attention entropy, inter-model consistency) and confirms that explanation quality scales with intrinsic task difficulty.

##### 4.3.2.4 Impact on clinical trust

When asked *post-hoc* whether seeing the Grad-CAM visualization changed their trust in the model’s prediction, 86.7% of raters reported increased trust (58 significantly increased, 72 slightly increased), 12.0% reported no change, 1.3% reported a slight decrease, and 0% reported a significant decrease. In the subset of incorrect predictions (*n* = 15 among the 150 evaluated samples), 9 cases led raters to decrease trust after reviewing the Grad-CAM (correctly flagging the error), while 6 cases maintained or increased trust despite the incorrect prediction (false reassurance). This suggests that Grad-CAM has moderate but imperfect utility for error detection and should complement rather than replace human judgment. Within this single evaluation, Grad-CAM was therefore associated with increased self-reported trust in the large majority of cases and did not induce false confidence in most of the incorrect-prediction cases examined; given the small sample of raters and the single-session design, this should be read as a preliminary signal rather than a general property of the method, and does not establish that Grad-CAM increases clinical trust in a generalizable sense.

##### 4.3.2.5 Expert-model attention alignment

To assess whether Grad-CAM’s highlighted regions matched the areas that experts consider diagnostically relevant, we asked raters to mentally identify the regions they would focus on for diagnosis, then estimate the overlap with the Grad-CAM heatmap on a 4-category scale (IoU > 0.7 = strong agreement; 0.5–0.7 = moderate; 0.3–0.5 = weak; < 0.3 = disagreement). Results showed 74.7% strong agreement, 18.7% moderate agreement, 5.3% weak agreement, and only 1.3% outright disagreement. This distribution indicates that in three-quarters of cases, the model’s attention closely aligned with expert reasoning, and in fewer than 2% of cases did the model focus on entirely irrelevant regions.

##### 4.3.2.6 Qualitative feedback themes

We coded all 450 free-text comments using a grounded theory approach to identify recurring themes. The most frequent positive comments were “accurate localization” (31.6%), “useful for confirmation” (28.4%), and “increased confidence in difficult cases” (25.6%). Critical feedback focused on two main issues: “sometimes too diffuse” (8.4%), referring to cases where attention spread across multiple cell regions without clear focus, and “background noise present” (5.3%), where low-level activations appeared in non-diagnostic areas. Representative positive comments included: “Grad-CAM correctly highlights the segmented nucleus in this neutrophil,” “The attention on eosinophilic granules is exactly what I look for,” and “This would help a junior resident learn where to focus.” Critical comments included: “Attention is spread across the entire cell, not specific enough,” and “Some activation on debris, but overall still useful.” No comments suggested that Grad-CAM misled the rater toward an incorrect diagnosis.

##### 4.3.2.7 Synthesis: clinical validity of grad-CAM

Taken together, the quantitative analysis (Section 4.3.1) and this expert evaluation provide converging, descriptive evidence that Grad-CAM’s explanations are clinically plausible and generally align with expert reasoning: (1) Grad-CAM intensity correlated with hand-annotated diagnostic features (r = 0.66–0.89, Section 4.3.1); (2) attention entropy distinguished correct from incorrect predictions (Section 4.3.1); (3) 86.7% of expert evaluations in this study reported increased self-reported trust after reviewing explanations; and (4) 74.7% of cases showed strong alignment between expert-identified and model-identified regions. As with any saliency-based explanation method, however, Grad-CAM cannot be shown to faithfully reflect the network’s internal decision process merely through correlation with plausible regions or expert agreement; it should be understood as an indicator of plausibility and a clinical communication aid, not as a mechanistic account of how the model arrived at its prediction. The analysis also characterizes Grad-CAM’s limitations: it struggles with staining artifacts, overlapping cells, and morphologically complex blast subtypes, which are precisely the scenarios where human oversight is most critical. This characterization of both strengths and failure modes provides a foundation for cautious clinical use: Grad-CAM may serve as a teaching tool, a decision-support aid for routine cases, and an error-flagging signal (via attention entropy), but should not replace expert judgment, and its explanations should not be interpreted as proof of the model’s underlying reasoning.

### 4.4 External validation

While high performance on the training dataset demonstrates the model’s learning capacity, external validation is essential to assess its generalizability to unseen clinical data. Models trained and evaluated on a single dataset often suffer from domain overfitting due to dataset-specific factors such as staining, imaging conditions, or patient demographics. External validation using an independent dataset provides a realistic estimate of model robustness, ensuring that the model can maintain its diagnostic performance when deployed in real-world settings across diverse laboratories and patient populations.

The Raabin-WBC dataset is a publicly available large-scale collection of peripheral blood smear images, containing over 14,000 manually annotated single-cell images from normal individuals. It includes five major leukocyte subtypes: Basophils, Eosinophils, Lymphocytes, Monocytes, and Neutrophils. Images were collected under varying conditions and from multiple devices, providing substantial inter-sample and inter-device variability. This inherent heterogeneity makes Raabin-WBC an ideal benchmark for evaluating domain generalization of the proposed UGDE model. Although it does not include blast cells (present in the training dataset), its inclusion of morphologically distinct normal leukocyte classes enables robust assessment of model performance on an unseen data distribution.

#### 4.4.1 Class mapping for external validation

The UGDE ensemble was trained to discriminate nine WBC subtypes (Basophils, Eosinophils, Lymphoblasts, Lymphocytes, Monocytes, Myeloblasts, Neutrophils (Band), Neutrophils (Segmented), and Normoblasts), whereas the Raabin-WBC dataset provides ground-truth labels for five morphologically normal leukocyte types (Basophils, Eosinophils, Lymphocytes, Monocytes, and Neutrophils) and, because it was collected from healthy individuals, contains no blast cells or nucleated red cells. To evaluate UGDE on Raabin-WBC, we defined the following correspondence between the two taxonomies. Four classes mapped one-to-one: Basophils, Eosinophils, Lymphocytes, and Monocytes. The two neutrophil maturation subtypes distinguished during training, Band and Segmented forms, were combined into Raabin-WBC’s single Neutrophil class by summing their predicted probabilities, since Raabin-WBC does not distinguish neutrophil maturation stage. The three classes with no counterpart in Raabin-WBC, Lymphoblasts, Myeloblasts, and Normoblasts, were excluded from the output space at evaluation time. Concretely, for each test image the original nine-class softmax output was restricted to the six retained logits (four singleton classes plus the two neutrophil subtypes), the two neutrophil probabilities were summed into a single Neutrophil probability, and the resulting five-class distribution was renormalized to sum to one. This renormalized five-class distribution was used both for the arg-max classification decision reported in Table 6 and Figure 8, and as the input to the conformal-prediction non-conformity scores reported in Section 4.5.2 and Table 7.

**Figure 8:**
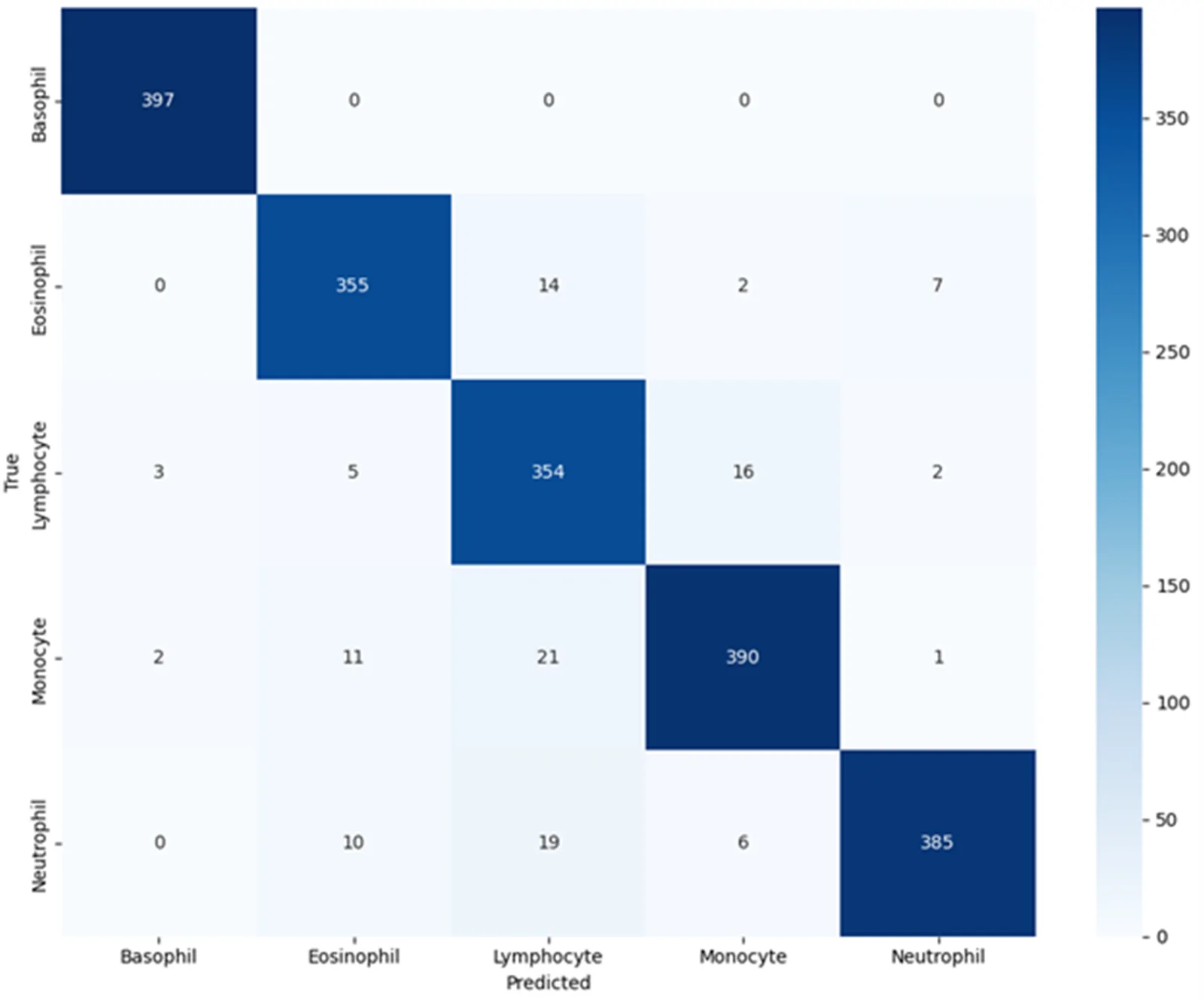
Confusion matrix of the UGDE model on the raabin-WBC external dataset. The model demonstrates high class-wise accuracy across all five leukocyte subtypes, with most predictions concentrated along the diagonal. Minimal misclassifications are observed primarily between morphologically similar classes such as lymphocytes, monocytes, and neutrophils, indicating strong generalization to unseen data.

**Table 6:** UGDE performance on the raabin-WBC dataset, achieving 94% overall accuracy with consistently high precision, recall, and F1-scores across all five leukocyte subtypes. .

Cell Type	Precision	Recall	F1-Score	Support
Basophil	0.99	1.00	0.99	397
Eosinophil	0.93	0.94	0.94	378
Lymphocyte	0.87	0.93	0.90	380
Monocyte	0.94	0.92	0.94	425
Neutrophil	0.97	0.92	0.94	420
Accuracy			0.94	2000
Macro avg	0.94	0.94	0.94	2000
Weighted avg	0.94	0.94	0.94	2000

**Table 7:** Summary of conformal prediction results.

Calibrated on 1,000 held-out Raabin-WBC images; evaluated on 1,000 images. Empirical coverage is compared with, but not equated to, nominal coverage. Finite-sample coverage guarantee (split conformal prediction) (56, 57): 1−*α* ≤ E[coverage] ≤ 1−α+1/(n_calib+1). At α = 0.05 with n_calib = 1,000, this guarantees coverage of at least 95.0%.							
α	Nominal	Empirical	Target met?	Empty (0)	Singleton (1)	Multi-class (≥2)	Mean size, lower bound
0.05	95.0%	94.6%	No (−0.4 pp)	54 (5.4%)	882 (88.2%)	64 (6.4%)	≥1.010
0.10	90.0%	90.2%	Yes (+0.2 pp)	98 (9.8%)	840 (84.0%)	62 (6.2%)	≥0.964
0.20	80.0%	80.4%	Yes (+0.4 pp)	196 (19.6%)	764 (76.4%)	40 (4.0%)	≥0.844

Table 6 summarizes the UGDE model’s performance on the Raabin-WBC dataset. The model achieved an overall accuracy of 94% across 2,000 test images, with macro-averaged precision, recall, and F1-score all at 0.94, indicating balanced performance across classes. Class-wise analysis revealed powerful results for Basophils (F1 = 0.99) and Neutrophils (F1 = 0.94), while Lymphocytes showed slightly lower precision (0.87) and F1-score (0.90), likely reflecting their higher morphological variability. These findings demonstrate that the UGDE model maintains high classification performance and generalizes effectively to unseen real-world data, supporting its potential utility in clinical hematology workflows.

Figure 8 presents the confusion matrix, which depicts the class-wise performance of the UGDE model on the Raabin-WBC dataset. The model correctly classified the majority of samples across all five leukocyte subtypes, with predictions predominantly concentrated along the diagonal, indicating strong overall discrimination capability. Basophils showed perfect classification, with all 397 samples correctly predicted and no misclassifications. Eosinophils also achieved high accuracy (355/378 correct), with a few samples misclassified as lymphocytes (14), neutrophils (7), or monocytes (2). Lymphocytes (354/380 correct) showed minor confusion primarily with monocytes (16) and eosinophils (5), which likely reflects morphological similarities in nuclear structure. Monocytes (390/425 correct) were occasionally misclassified as lymphocytes (21) or eosinophils (11). Neutrophils (385/420 correct) had small misclassification rates into lymphocytes (19) and eosinophils (10).

These patterns align with the per-class metrics, where basophils achieved the highest precision and recall, while lymphocytes and monocytes exhibited slightly higher misclassification rates. Overall, the confusion matrix confirms that UGDE maintained high class-wise reliability and balanced performance on an unseen dataset, demonstrating good generalization across morphologically diverse leukocyte types.

Figure 9 presents the reliability (calibration) curve of the UGDE ensemble on the Raabin-WBC external dataset. The *x*-axis represents the mean predicted confidence, and the *y*-axis shows the fraction of correct predictions within each confidence bin. The orange dashed line represents a perfectly calibrated model where predicted confidence matches the observed accuracy. The UGDE ensemble exhibits reasonable calibration, with most plotted points closely following the diagonal, especially at higher confidence ranges (≥0.6). Predictions made with high confidence (0.8–1.0) correspond to high observed accuracy (≈0.85–1.0), indicating trustworthy confidence estimates for strong predictions. At lower confidence levels (<0.5), the curve lies below the diagonal, showing slight under confidence, which is a safer failure mode than overconfidence. Overall, these results demonstrate that the UGDE model produces well-calibrated probability estimates on unseen data, which is essential for uncertainty-aware decision support in clinical practice.

**Figure 9:**
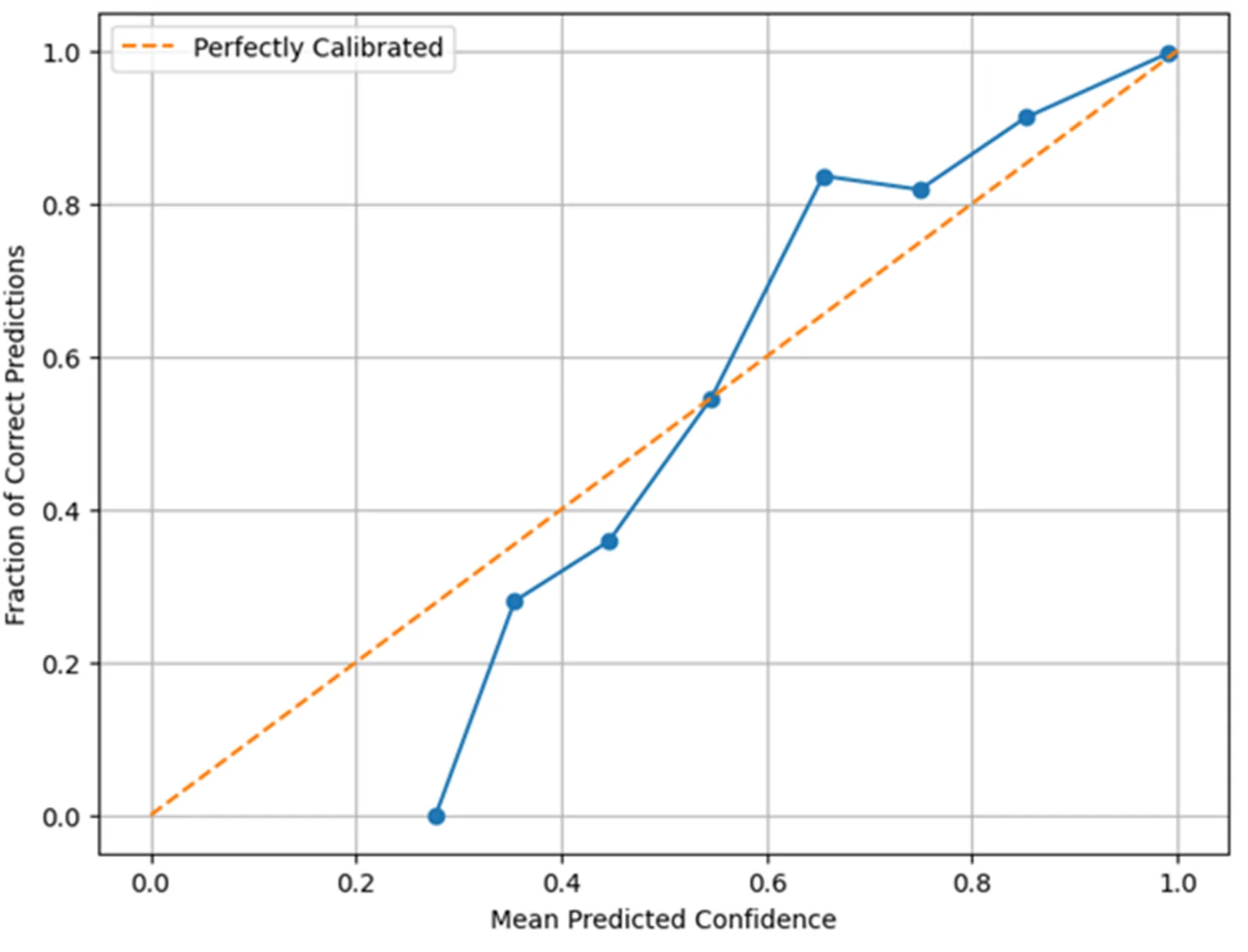
Reliability diagram of the UGDE ensemble on the external raabin-WBC dataset. The blue curve shows the fraction of correct predictions as a function of predicted confidence, while the orange dashed line represents perfect calibration. The UGDE ensemble closely follows the diagonal trend, indicating well-calibrated probability estimates that align predicted confidence with observed accuracy.

#### 4.4.2 Conformal prediction on external validation

To provide rigorous, distribution-free uncertainty quantification on the external dataset, we applied the conformal prediction framework (56, 57) to the Raabin-WBC dataset using a re-calibration strategy that ensures the exchangeability assumption is satisfied within the external domain. The Raabin-WBC dataset (n = 2,000) was divided equally into an external calibration set (n = 1,000) and an external evaluation set (n = 1,000). Non-conformity scores were recomputed using external calibration samples, and prediction sets Ĉ*α*(x) were constructed for the evaluation set at three significance levels (*α* ∈ {0.05, 0.10, 0.20}).

Results are summarized in Table 7. The *α* = 0.10 and *α* = 0.20 levels meet their theoretical guarantee (empirical coverage of 90.2% and 80.4%, against nominal targets of 90.0% and 80.0%); the *α* = 0.05 level falls 0.4 percentage points short (94.6% against a nominal 95.0%). In deployment, empty and multi-class prediction sets are treated as review triggers rather than errors: the former indicates that no class met the confidence threshold, and the latter indicates genuine ambiguity between two or more classes, both warranting pathologist adjudication.

A key finding distinguishes the external analysis from the internal analysis: a proportion of prediction sets had size ≥ 2 on the external dataset (6.4% at *α* = 0.05), whereas no multi-class prediction sets were observed on the internal test set. Inspection of these cases revealed that they arise predominantly at lymphocyte/monocyte and monocyte/neutrophil decision boundaries, which are the same cell pairs that produced the highest misclassification rates in the external confusion matrix. This behavior is clinically meaningful: the model correctly flags morphologically ambiguous cells as requiring expert review rather than forcing a potentially erroneous singleton prediction. The mean prediction set size at *α* = 0.05 was at least 1.010 on the external dataset (Table 7), necessarily higher than the internal test set, where the absence of any multi-class sets caps the mean at 1.0, reflecting the additional uncertainty introduced by domain shift and the emergence of multi-class sets at the lymphocyte/monocyte and monocyte/neutrophil boundaries.

A sensitivity analysis was also conducted by transferring the internal calibration threshold q^*α* (derived from the primary training dataset) directly to all 2,000 Raabin-WBC samples without re-calibration. This yielded empirical coverage of 94.1%, 89.8%, and 79.7% at *α* = 0.05, 0.10, and 0.20, respectively, all within 1.0% of the re-calibrated values. While this approach does not carry a theoretical coverage guarantee in the presence of distribution shift, the close agreement between transfer and re-calibrated coverage confirms that the model’s non-conformity scores are portable across imaging domains and that the uncertainty representation is not overfitted to the primary dataset.

Figure 10 illustrates representative Grad-CAM heatmaps with associated predictive entropy scores generated by the UGDE model on the Raabin-WBC external dataset. For each cell type (Basophil, Eosinophil, Lymphocyte, Monocyte, and Neutrophil), the original input image is displayed alongside the Grad-CAM overlay, which highlights the discriminative regions used by the model, along with the predicted class and its corresponding entropy value.

**Figure 10:**
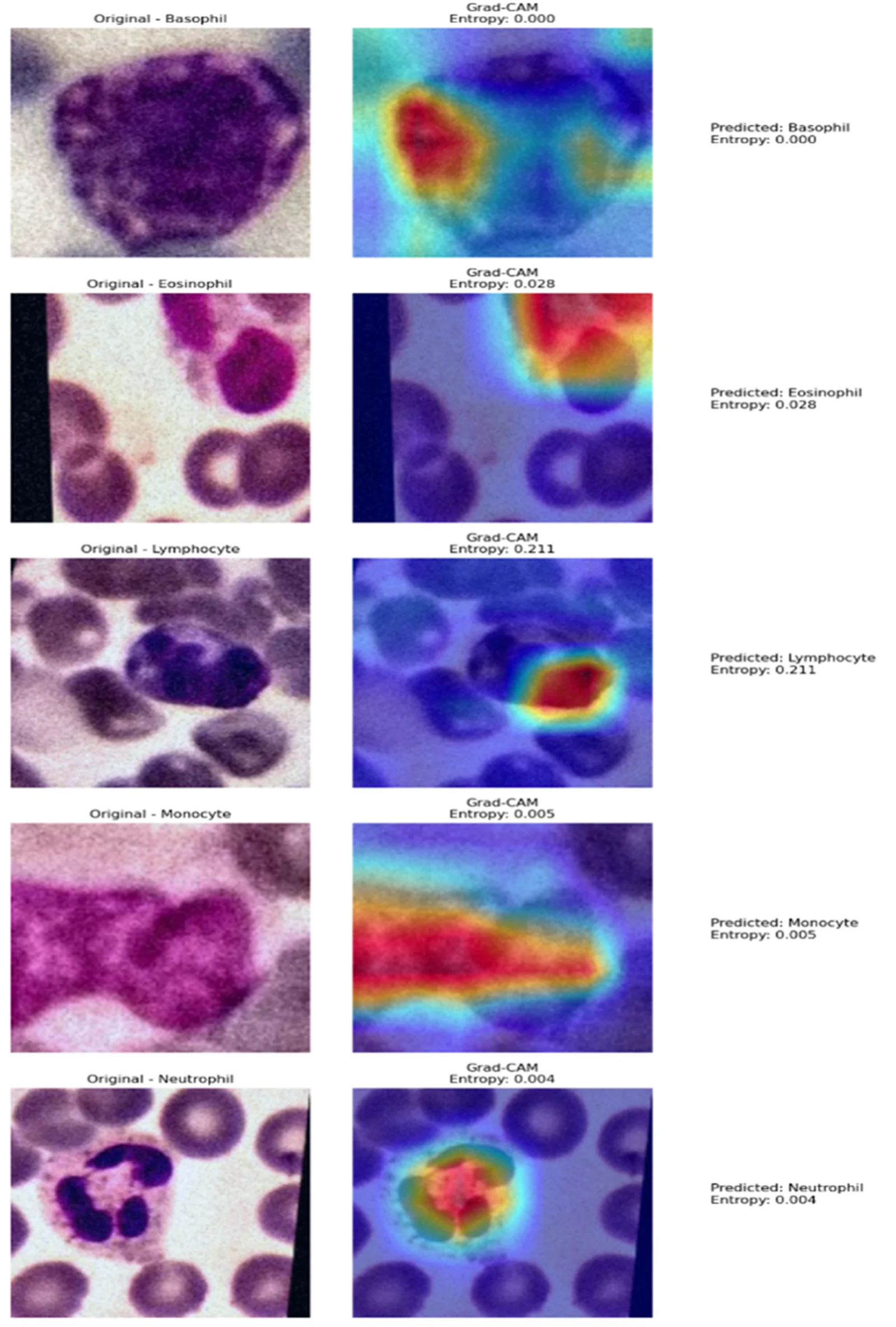
Grad-CAM visualizations with predictive entropy scores for representative raabin-WBC samples. For each leukocyte subtype (Basophil, Eosinophil, Lymphocyte, Monocyte, Neutrophil), the UGDE model highlights morphologically relevant nuclear and cytoplasmic regions while yielding low entropy values for confident predictions, demonstrating interpretable and uncertainty-aware performance on external data. Cell images from “Raabin-WBC: a large free access dataset of white blood cells from normal peripheral blood” dataset by View ORCID ProfileZahra Mousavi Kouzehkanan, Sepehr Saghari, Eslam Tavakoli, Peyman Rostami, Mohammadjavad Abaszadeh, Farzaneh Mirzadeh, Esmaeil Shahabi Satlsar, Maryam Gheidishahran, Fatemeh Gorgi, Saeed Mohammadi and Reshad Hosseini, licensed under CC BY-NC.

Across all classes, Grad-CAM consistently highlighted morphologically relevant regions, particularly nuclear and cytoplasmic structures, confirming that the model’s decision-making is grounded in biologically meaningful features. The associated entropy scores were generally very low for correctly classified cells (Basophil: 0.000, Monocyte: 0.005, Neutrophil: 0.004), reflecting high model confidence. Slightly higher entropy was observed for classes with more intra-class variability, such as Lymphocytes (0.211) and Eosinophils (0.028), indicating moderate uncertainty. Overall, these visualizations show that UGDE not only generalizes well to unseen data but also produces transparent and confidence-aware predictions, enabling users to interpret which morphological regions contributed to the classification and to recognize predictions made with lower confidence.

### 4.5 Descriptive comparison of class-wise F1 scores

Table 8 presents a descriptive comparison of F1 scores across the nine cell classes. UGDE achieved the highest mean class-wise F1 score (0.990 ± 0.007), followed by ShuffleNetV2 (0.989 ± 0.008), MobileNetV3-Large (0.984 ± 0.007), and EfficientNet-B0 (0.984 ± 0.013). All models had a median F1 score of 0.99. UGDE scores ranged from 0.98 to 1.00, compared with 0.97–0.99 for MobileNetV3-Large, 0.96–1.00 for EfficientNet-B0, and 0.98–1.00 for ShuffleNetV2.

**Table 8:** Descriptive comparison of class-wise F1 scores for individual models and the UGDE ensemble.

Model	Mean class-wise F1 ± SD	Median	Range	UGDE higher/equal/lower
MobileNetV3-Large	0.984 ± 0.007	0.99	0.97–0.99	5/4/0
EfficientNet-B0	0.984 ± 0.013	0.99	0.96–1.00	4/5/0
ShuffleNetV2	0.989 ± 0.008	0.99	0.98–1.00	1/8/0
UGDE	0.990 ± 0.007	0.99	0.98–1.00	—

Based on the reported class-wise values, UGDE achieved higher F1 scores in five, four, and one classes compared with MobileNetV3-Large, EfficientNet-B0, and ShuffleNetV2, respectively, and equal scores in the remaining classes. No lower class-wise F1 scores were observed for UGDE at the reported precision. The standard deviations represent variation across cell classes, not uncertainty across patients or repeated training runs. These findings indicate modest numerical improvements in class-wise performance but do not establish statistical significance or patient-level superiority.

## 5 Discussion

Our results demonstrate that it is feasible to achieve near-perfect classification of white blood cells into a broad range of categories using deep learning, provided a sufficiently large and high-quality dataset is available. The base models we trained already performed exceptionally (98%–99% accuracy), reflecting both the power of modern CNNs and the fact that many WBC types are visually distinct. The slight edge of ShuffleNetV2 in our experiments may be attributed to its architecture being particularly well-suited to the dataset, or simply to random variation; all three models were quite close in performance. By combining them (UGDE), we essentially garnered the “wisdom of models,” which yielded a marginal yet consistent improvement and, importantly, smoothed out quirks of individual networks.

### 5.1 Uncertainty quantification

One of the primary benefits of our UGDE approach is the ability to know when the model might be wrong. This is critical in medical AI; an overly confident error can be dangerous. Our ensemble’s uncertainty estimates correlated well with complex cases (e.g., ambiguous morphology or image artifacts). For instance, the model appropriately had lower confidence in atypical lymphocytes that resembled blasts. In a clinical setting, these uncertainty flags could prompt a manual review. This is akin to a junior pathologist asking for a senior opinion when unsure. Notably, the deep ensemble with MC dropout provides a form of Bayesian model averaging (20) which is theoretically grounded in uncertainty estimation. An alternative approach could be a fully Bayesian neural network or test-time augmentation, but MC dropout proved to be a simple and effective technique in our work. The calibration results (ECE ∼1% after scaling) are promising; our model’s probability outputs can be interpreted as true likelihoods.

While Monte Carlo (MC) dropout provides a Bayesian approximation to model uncertainty by sampling multiple stochastic forward passes, its estimates depend on architectural choices and the number of runs. In contrast, conformal prediction (CP) is a distribution-free approach that guarantees user-defined coverage irrespective of the underlying model. Our results show that both methods yield consistent conclusions: UGDE predictions are generally well-calibrated and confident on most WBC images, with ambiguous cases correctly flagged by increased uncertainty. Importantly, CP complements MC dropout by offering formal statistical guarantees on coverage, while maintaining practical utility through small set sizes (≈1.0 in nearly all cases). Together, these two methods provide complementary perspectives, probabilistic confidence (MC dropout) and guaranteed reliability (CP), which strengthen the case for deploying UGDE in clinical hematology workflows.

Few prior WBC classification studies have reported on calibration or uncertainty; we hope our findings encourage others to incorporate these aspects, as they can improve patient safety (for example, by only acting on automated results above a certain confidence threshold and deferring to human expertise otherwise).

### 5.2 Interpretability and trust

The Grad-CAM visualizations provided a qualitative assurance of our model’s validity. They showed that the network is focusing on cell features that a hematologist would deem important, a reassuring fact that builds trust in the system’s reasoning process. In contrast, if the heatmaps had highlighted irrelevant background or consistently focused on an artifact (such as the slide’s staining edges or debris), it would indicate that the model might be exploiting spurious correlations, which would be concerning. We did not observe such behavior in our model. On the contrary, as shown, the attention maps overlapped with nuclei, granules, and cytoplasm of cells, which are precisely where the diagnostic information lies. This interpretability not only helps end-users trust the model but can also aid developers in debugging. For example, if a class’s heatmaps appear to be off, we might investigate the training data for that class or the network’s layer representations. In our case, the interpretability analysis bolstered our confidence that the model had learned genuine morphological features of WBCs.

### 5.3 Comparison to prior work

Compared to previous WBC classification studies, our approach is among the first to focus on uncertainty and interpretability in addition to raw performance. Many earlier works report high accuracies on similar tasks (4–12), but they do not indicate how the model might behave in less clear-cut cases or how it makes decisions. Our ensemble’s performance (99% accuracy, 0.99 macro-F1) is at least on par with state-of-the-art results on similar 5-class problems and extends it to a more challenging 9-class scenario, including blasts. For example, Dhibar (32) reported an accuracy of ∼98% using an ensemble on five classes; our results on nine classes match that level, demonstrating good generalization. This can be partly attributed to the rich training data, the Bodzás et al. dataset, which underscores the importance of open medical data for advancing AI. Our use of efficient networks (MobileNet, etc.) also demonstrates that one does not necessarily need extremely deep models (such as ResNet-101 or DenseNet-201, as used in some works) to achieve outstanding performance on this task, especially with transfer learning. Lighter models can suffice and are preferable for deployment.

A comparative analysis of recent state-of-the-art approaches for white blood cell (WBC) classification is presented in Table 9. Several studies have reported impressive accuracies using diverse architectures and datasets; for example, Asghar et al. achieved up to 99.91% accuracy using transfer learning with ResNet-101, InceptionV3, and DenseNet-201 on the PBC dataset, while Khan et al. reported accuracies above 99% by integrating a dual attention mechanism into a CNN on the PBC, LISC, and Raabin-WBC datasets. Other works, such as those by Anand et al. and Saikia et al., also showed competitive performance (97%–98%) using conventional CNNs and SVM-based pipelines, respectively, albeit on fewer classes or smaller datasets. However, most of these methods either relied on large pre-trained networks with high computational costs or lacked mechanisms for uncertainty estimation and interpretability, which are crucial for clinical adoption. In contrast, our proposed Uncertainty-Guided Deep Ensemble (UGDE) model achieved approximately 99% accuracy with uniformly high precision, recall, and F1-scores (0.98–1.00) across nine clinically relevant WBC subtypes using the high-resolution dataset of Bodzás et al. Significantly, UGDE enhances predictive performance with calibrated probability estimates and Grad-CAM–based visual explanations, facilitating the reliable identification of uncertain predictions. This combination of accuracy, awareness of uncertainty, and interpretability distinguishes our approach from existing models, making it more suitable for real-world deployment in hematology workflows.

**Table 9:** Comparison of recent white blood cell (WBC) classification methods with the proposed uncertainty-guided deep ensemble (UGDE).

Study	Model/method	Dataset(s) used	# classes	Accuracy (%)	Other metrics	Reported limitation
Asghar et al. (2024) (4)	Transfer learning (ResNet-101, InceptionV3, DenseNet-201)	LISC, Kaggle, PBC	10	98.79/99.68/99.91	—	High execution time
Patil et al. (2021) (26)	Deep CNN feature extraction + Canonical Correlation Analysis (CCA) fusion	LISC, BCCD	5	95.89	Precision/Recall/F1 ≈ 96%–98%	Not end-to-end trainable; limited generalization on large-scale unseen data
Sharvani et al. (2023). (58)	Deep transfer learning (VGG16, ResNet50, InceptionV3, DenseNet201)	Kaggle blood cell images dataset with 12500 images	4	92	Precision/Recall/F1 ≈ 85%	High computational cost; lacks uncertainty and interpretability
Khan et al. (2024) (3)	CNN + Dual Attention	PBC, LISC, Raabin-WBC	5	99.83/99.35/99.60	—	Computational complexity, lacks uncertainty and interpretability
Anand et al. (2024) (5)	Custom CNN	Kaggle (12,444 images)	4	97.98	Precision 97.97%	No attention mechanisms, lacks uncertainty and interpretability
Zonyfar et al. (2024) (6)	Convolutional Attention BLSTM (CAB-Net)	Small custom dataset	8	98.29	Precision/Recall/F1 98%	Insufficient dataset size, lacks uncertainty, and interpretability
Fathy et al. (2023) (7)	ResNet101 + BBCO + SVM	Raabin-WBC	5	99.21	—	No external validation, lacks uncertainty and interpretability
Saikia et al. (2024) (8)	WOA-SVM + handcrafted features	ALL-IDB1	2 (ALL vs. normal)	98.42	—	High computational time, lacks uncertainty, and interpretability
Rivas et al. (2023) (9)	Meta-learning-based base-model selection	Raabin-WBC, BCCD, UACH	5	98.29	—	Computational overhead, lacks uncertainty, and interpretability.
Prasad et al. (2024). (11)	Deep U_ClusterNet (segmentation + clustering)	Custom dataset	5	98.2	Precision 95.7/Recall 99.8/Spec 98.3/DSC 95.1	High computational resources required, lacks uncertainty and interpretability
Leng et al. (2023) (12)	DETR with Vision Transformer + deformable attention	Modified Raabin leukocyte	5	—	Mean Average Precision 96.1%	Computational complexity lacks uncertainty and interpretability
Proposed UGDE	Uncertainty-Guided Deep Ensemble (MobileNetV3 + EfficientNet-B0 + ShuffleNetV2 + MC Dropout + Grad-CAM)	Bodzás et al. 2023 (16,027 WBC images from 78 patients)	9	**∼99**.**0**	Precision/Recall/F1 ≈ 0.98–1.00, well-calibrated probabilities, uncertainty estimation	A slight performance drop on ambiguous classes necessitates ensemble inference.

Bold values indicate results of the proposed UGDE model.

Collectively, this comparative analysis highlights that while many existing WBC classification models have achieved high accuracy, they often overlook two critical aspects essential for safe clinical deployment: quantified uncertainty and transparent interpretability. Our UGDE framework addresses these gaps by integrating multiple lightweight CNNs with Monte Carlo dropout–based uncertainty estimation and Grad-CAM visual explanations, thereby offering not only state-of-the-art accuracy (∼99%) but also reliable confidence calibration and biologically meaningful decision insights. This synergy of performance, reliability, and explainability positions UGDE as a promising and trustworthy decision-support tool for hematological diagnostics, bridging the gap between experimental deep learning models and practical clinical applications.

### 5.4 Clinical impact

With approximately 99% classification accuracy on pre-cropped single-cell images, the model demonstrates strong performance on the nine-class WBC identification task evaluated in this study; a direct, prospective comparison against practicing morphologists’ accuracy on the same cases was not performed here and would be needed to substantiate a comparison to expert-level reliability. The model’s consistency and speed on this task are notable advantages. Integrated with a cell-detection and segmentation front end and validated on whole-slide images neither of which was evaluated in this study (see Future Directions) such a system could in principle analyze a microscope slide containing hundreds of cells and support an automated differential count, which could aid hematology laboratories, especially in resource-limited settings where experienced morphologists are few; this remains a target for future work rather than a demonstrated capability of the present system. The conformal-prediction uncertainty framework is designed to flag low-confidence predictions for human review (for example, atypical or morphologically ambiguous cells within the nine trained classes); its behavior under image-quality degradation or other out-of-distribution conditions was not exhaustively evaluated here (see Limitations), and should not yet be assumed to extend to scenarios such as poor smear quality. Ultimately, the tool is intended not to replace a pathologist but to augment expert workflow, potentially allowing routine cases to be handled with less manual review while flagging atypical or low-confidence cases for expert attention, pending the prospective validation described in Future Directions. The interpretability provided by Grad-CAM could also serve as an educational aid: when the model misclassifies a case and the heatmap highlights non-diagnostic regions, a specialist can use this as a starting point for reviewing the model’s training data or failure modes, though, as noted in the Limitations, Grad-CAM is a *post-hoc* saliency method and its explanations do not establish a causal account of the model’s internal computation. Moreover, explainability is increasingly emphasized in regulatory guidance for AI in medicine, and interpretability tools such as Grad-CAM are one component of what such guidance typically expects, though this study does not constitute or substitute for a regulatory submission or review.

## 6 Conclusion

In this study, we proposed an Uncertainty-Guided Deep Ensemble (UGDE) framework for white blood cell classification that integrates complementary lightweight CNN architectures, Monte Carlo dropout–based uncertainty estimation, conformal prediction, and Grad-CAM interpretability. The proposed approach achieved near 99% accuracy across nine morphologically distinct leukocyte subtypes on a large high-resolution dataset, while also producing well-calibrated probability estimates and clinically plausible visual explanations, supported by a preliminary expert evaluation Beyond raw classification performance, the integration of uncertainty quantification and explainability provides an additional safety layer that is essential for high-stakes medical decision support systems.

Importantly, our results demonstrate that high accuracy alone is insufficient for clinical translation; reliability and interpretability are equally critical. The ensemble framework reduced class-wise variance, improved calibration (ECE reduced to <1% after scaling), and maintained strong generalization on an external dataset, supporting the robustness of the proposed method under domain shift. These findings suggest that uncertainty-aware ensembles represent a promising direction for trustworthy AI deployment in hematology workflows.

### 6.1 Limitations

Despite the strong performance, several limitations must be acknowledged.

First, although the dataset is large and balanced through augmentation, it originates from a limited number of centers and staining protocols. Real-world hematology laboratories may exhibit broader variability in staining intensity, microscope optics, imaging devices, and patient demographics. Such distribution shifts may affect performance, particularly for morphologically subtle subtypes.

Second, the current taxonomy includes nine clinically relevant classes but does not encompass rarer or intermediate maturation stages (e.g., promyelocytes, plasma cells, atypical lymphocytes). Expanding to a more granular hematological ontology may introduce additional class overlap and increase uncertainty.

Third, the computational overhead of the ensemble with Monte Carlo dropout is non-trivial. Performing multiple stochastic forward passes across three models increases inference time, which may limit real-time deployment without optimization, pruning, or model distillation.

Fourth, although uncertainty estimation was validated statistically, epistemic uncertainty under severe out-of-distribution (OOD) scenarios was not exhaustively evaluated. True OOD detection in hematological imaging remains an open research challenge.

Finally, while Grad-CAM provides visual interpretability, it remains a *post-hoc* explanation method and does not guarantee causal faithfulness. More inherently interpretable model architectures may provide stronger transparency guarantees.

### 6.2 Future directions

Future work should focus on multi-center validation across geographically diverse laboratories to rigorously evaluate robustness under real-world variability. Domain adaptation techniques and stain normalization strategies may further enhance generalization. Expanding the classification framework to include additional immature and rare hematological subtypes will improve clinical applicability, particularly in complex leukemia diagnostics. Integration with whole-slide image analysis pipelines could enable automated differential counts directly from full smear images rather than pre-cropped cells.

From an uncertainty modeling perspective, comparison with advanced approaches such as evidential deep learning, Bayesian neural networks, and out-of-distribution detection frameworks would provide deeper insight into reliability under extreme uncertainty conditions. Additionally, risk-coverage optimization strategies could allow deployment under user-defined safety thresholds. To improve efficiency, future studies may explore ensemble distillation or lightweight transformer–CNN hybrid architectures that retain uncertainty-awareness while reducing computational cost. Finally, prospective user studies involving practicing hematologists are necessary to assess how uncertainty flags and visual explanations influence diagnostic confidence, workflow efficiency, and trust in AI-assisted decision-making.

Overall, the proposed UGDE framework advances white blood cell classification beyond conventional accuracy-driven approaches by embedding statistical reliability and interpretability into the core architecture. By explicitly modeling uncertainty and providing biologically meaningful explanations, this work contributes toward the development of clinically trustworthy AI systems that complement, rather than replace, expert judgment in hematological diagnostics.

## Data Availability

The original contributions presented in the study are included in the article/supplementary material, further inquiries can be directed to the corresponding authors.

## References

[1] Bodzas A Kodytek P Zidek J. A high-resolution, large-scale dataset of pathological and normal white blood cells. Sci Data. (2023) 10(1):466. 10.1038/s41597-023-02378-7 37468490 PMC10356748

[2] Tamang T Baral S Paing MP. Classification of white blood cells: a comprehensive study using transfer learning based on convolutional neural networks. Diagnostics. (2022) 12(12):2903. 10.3390/diagnostics12122903 36552910 PMC9777002

[3] Khan S Sajjad M Abbas N Escorcia-Gutierrez J Gamarra M Muhammad K. Efficient leukocytes detection and classification in microscopic blood images using convolutional neural network coupled with a dual attention network. Comput Biol Med. (2024) 174:108146. 10.1016/j.compbiomed.2024.108146 38608320

[4] Asghar R Kumar S Hynds P. Automatic classification of 10 blood cell subtypes using transfer learning via pre-trained convolutional neural networks. Inform Med Unlocked. (2024) 49:101542. 10.1016/j.imu.2024.101542

[5] Anand V Gupta S Koundal D Alghamdi WY Alsharbi BM. Deep learning-based image annotation for leukocyte segmentation and classification of blood cell morphology. BMC Med Imaging. (2024) 24(1):83. 10.1186/s12880-024-01254-z 38589793 PMC11003052

[6] Zonyfar C Kim JD. CAB-net: convolutional attention BLSTM network for enhanced leukocyte classification. Int J Imaging Syst Technol. (2024) 34(2):e23065. 10.1002/ima.23065

[7] Fathy KA Yaseen HK Abou-Kreisha MT ElDahshan KA. A novel meta-heuristic optimization algorithm in white blood cells classification. Comput., Mater. Continua. (2023) 75(1):1527–45. 10.32604/cmc.2023.036322

[8] Saikia R Sarma A Shuleenda Devi S. Optimized support vector machine using whale optimization algorithm for acute lymphoblastic leukemia detection from microscopic blood smear images. SN Comput Sci. (2024) 5(5):439. 10.1007/s42979-024-02822-4

[9] Rivas-Posada E Chacon-Murguia MI. Automatic base-model selection for white blood cell image classification using meta-learning. Comput Biol Med. (2023) 163:107200. 10.1016/j.compbiomed.2023.107200 37393786

[10] Veeraiah N Alotaibi Y Subahi AF. MayGAN: mayfly optimization with generative adversarial network-based deep learning method to classify leukemia form blood smear images. Comput. Syst. Sci. Eng. (2023) 46(2):2039–58. 10.32604/csse.2023.036985

[11] Prasad PK Reddy ES Sekharaiah KC. Deep U_ClusterNet: automatic deep clustering based segmentation and robust cell size determination in white blood cell. Multimed Tools Appl. (2024) 83(9):25923–49. 10.1007/s11042-023-16521-4

[12] Leng B Wang C Leng M Ge M Dong W. Deep learning detection network for peripheral blood leukocytes based on improved detection transformer. Biomed Signal Process Control. (2023) 82:104518. 10.1016/j.bspc.2022.104518

[13] Mehmood S Shahzad T Zubair M Khan FM Khan MA Ouahada K. Improved leukocyte classification in bone marrow cytology using convolutional neural network with contrast enhancement. Sci Rep. (2025) 15(1):30466. 10.1038/s41598-025-12207-z 40830111 PMC12365091

[14] Hussain SS Shah PM Dawood H Degang X Alshamayleh A Khan MA. A swin transformer and CNN fusion framework for accurate Parkinson disease classification in MRI. Sci Rep. (2025) 15(1):15117. 10.1038/s41598-025-93671-5 40301455 PMC12041535

[15] Alqaraleh M Khleifat KM Abu Hajleh MN Farah HS Ahmed KAA. Fungal-mediated silver nanoparticle and biochar synergy against colorectal cancer cells and pathogenic bacteria. Antibiotics. (2023) 12(3):597. 10.3390/antibiotics12030597 36978464 PMC10044691

[16] Alhussan AA Abdelhamid AA Towfek SK Ibrahim A Abualigah L Khodadadi N. Classification of breast cancer using transfer learning and advanced al-biruni earth radius optimization. Biomimetics. (2023) 8(3):270. 10.3390/biomimetics8030270 37504158 PMC10377265

[17] Rustam F Al-Shamayleh AS Shafique R Obregon SA Iglesias RC Gonzalez JP. Enhanced detection of diabetes mellitus using novel ensemble feature engineering approach and machine learning model. Sci Rep. (2024) 14(1):23274. 10.1038/s41598-024-74357-w 39375469 PMC11458802

[18] Mehmood S Ghazal TM Khan MA Zubair M Naseem MT Faiz T. Malignancy detection in lung and colon histopathology images using transfer learning with class selective image processing. IEEE Access. (2022) 10:25657–68. 10.1109/ACCESS.2022.3150924

[19] Kim D Sundling KE Virk R Thrall MJ Alperstein S Bui MM. Digital cytology part 1: digital cytology implementation for practice: a concept paper with review and recommendations from the American society of cytopathology digital cytology task force. J Am Soc Cytopathol. (2024) 13(2):86–96. 10.1016/j.jasc.2023.11.006 38158316

[20] Gal Y Ghahramani Z. Dropout as a Bayesian approximation: representing model uncertainty in deep learning. In: Balcan MF Weinberger KQ, editors. Proc. 33rd Int. Conf. Mach. Learn. PMLR, vol. 48. (2016).

[21] Hehr M Sadafi A Matek C Lienemann P Pohlkamp C Haferlach T. Explainable AI identifies diagnostic cells of genetic AML subtypes. PLOS Digit Health. (2023) 2(3):e0000187. 10.1371/journal.pdig.0000187 36921004 PMC10016704

[22] Rudin C. Stop explaining black box machine learning models for high stakes decisions and use interpretable models instead. Nat Mach Intell. (2019) 1(5):206–15. 10.1038/s42256-019-0048-x 35603010 PMC9122117

[23] Adjouadi M Zong N Ayala M. Multidimensional pattern recognition and classification of white blood cells using support vector machines. Part Part Syst Charact. (2005) 22(2):107–18. 10.1002/ppsc.200400888

[24] Singh I Singh NP Singh H Bawankar S Ngom A. Blood cell types classification using CNN. In: Rojas I Valenzuela O Rojas F Herrera LJ Ortuño F, editors. Bioinformatics and Biomedical Engineering (IWBBIO 2020). Lecture Notes in Computer Science, vol. 12108. Cham: Springer International Publishing (2020). p. 727–38. 10.1007/978-3-030-45385-5_65

[25] Ma L Shuai R Ran X Liu W Ye C. Combining DC-GAN with ResNet for blood cell image classification. Med Biol Eng Comput. (2020) 58(6):1251–64. 10.1007/s11517-020-02163-3 32221797

[26] Patil AM Patil MD Birajdar GK. White blood cells image classification using deep learning with canonical correlation analysis. Irbm. (2021) 42(5):378–89. 10.1016/j.irbm.2020.08.005

[27] Şengür A Akbulut Y Budak Ü Cömert Z. White blood cell classification based on shape and deep features. In: 2019 International Artificial Intelligence and Data Processing Symposium (IDAP). Piscataway, NJ: IEEE (2019). p. 1–4.

[28] Chen Y Chen Z An Y Lu C Qiao X. DAFFNet: a dual attention feature fusion network for classification of white blood cells. Biomed Signal Process Control. (2025) 106:107699. 10.1016/j.bspc.2025.107699

[29] Abou Ali M Dornaika F Arganda-Carreras I. White blood cell classification: convolutional neural network (CNN) and vision transformer (ViT) under medical microscope. Algorithms. (2023) 16(11):525. 10.3390/a16110525

[30] Park S Cho H Woo BM Lee SM Bae D Balint A. A large multi-focus dataset for white blood cell classification. Sci Data. (2024) 11(1):1106. 10.1038/s41597-024-03938-1 39384810 PMC11464576

[31] Lakshminarayanan B Pritzel A Blundell C. Simple and scalable predictive uncertainty estimation using deep ensembles. Adv Neural Inf Process Syst. (2017) 30:6402–13.

[32] Dhibar S. DCENWCNet: A Deep CNN Ensemble Network for White Blood Cell Classification with LIME-Based Explainability. arXiv preprint arXiv:2502.05459 (2025).

[33] Panthakkan A Anzar SM Mansoor W Al Ahmad H. A new frontier in hematology: robust deep learning ensembles for white blood cell classification. Biomed Signal Process Control. (2025) 100:106995. 10.1016/j.bspc.2024.106995

[34] Li M Wang J Fang S Yang L Liu X Yun H. Research and optimization of white blood cell classification methods based on deep learning and Fourier ptychographic microscopy. Sensors. (2025) 25(9):2699. 10.3390/s25092699 40363138 PMC12074126

[35] Gour M Jain S. Uncertainty-aware convolutional neural network for COVID-19 x-ray images classification. Comput Biol Med. (2022) 140:105047. 10.1016/j.compbiomed.2021.105047 34847386 PMC8609674

[36] Zahari R Cox J Obara B. Uncertainty-aware image classification on 3D CT lung. Comput Biol Med. (2024) 172:108324. 10.1016/j.compbiomed.2024.108324 38508053

[37] Abdar M Fahami MA Rundo L Radeva P Frangi AF Acharya UR. Deep hierarchical attentive multilevel fusion model with uncertainty quantification for medical image classification. IEEE Trans Ind Inform. (2022) 19(1):274–85. 10.1109/TII.2022.3168887

[38] Ju L Wang X Wang L Mahapatra D Zhao X Zhou Q. Improving medical images classification with label noise using dual-uncertainty estimation. IEEE Trans Med Imaging. (2022) 41(6):1533–46. 10.1109/TMI.2022.3141425 34995185

[39] Valen J Balki I Mendez M Qu W Levman J Bilbily A. Quantifying uncertainty in machine learning classifiers for medical imaging. Int J Comput Assist Radiol Surg. (2022) 17(4):711–8. 10.1007/s11548-022-02578-3 35278156

[40] Senousy Z Abdelsamea MM Gaber MM Abdar M Acharya UR Khosravi A. MCUa: multi-level context and uncertainty aware dynamic deep ensemble for breast cancer histology image classification. IEEE Trans Biomed Eng. (2021) 69(2):818–29. 10.1109/TBME.2021.3107446 34460359

[41] Arco JE Ortiz A Ramirez J Martinez-Murcia FJ Zhang YD Gorriz JM. Uncertainty-driven ensembles of multi-scale deep architectures for image classification. Inf Fus. (2023) 89:53–65. 10.1016/j.inffus.2022.08.010

[42] Rahaman R Thiery AH. Uncertainty quantification and deep ensembles. Adv Neural Inf Process Syst. (2021) 34:20063–75.

[43] Hirsch R Goldberger J. A conformalized learning of a prediction set with applications to medical imaging classification. In: 2024 IEEE International Symposium on Biomedical Imaging (ISBI). Piscataway, NJ: IEEE (2024). p. 1–5.

[44] Selvaraju RR Cogswell M Das A Vedantam R Parikh D Batra D. Grad-cam: visual explanations from deep networks via gradient-based localization. In: Proceedings of the IEEE International conference on Computer vision. Piscataway, NJ: IEEE (2017). p. 618–26. 10.1109/ICCV.2017.74

[45] Chossegros M Delhommeau F Stockholm D Tannier X. Improving the generalizability of white blood cell classification with few-shot domain adaptation. J Pathol Inform. (2024) 15:100405. 10.1016/j.jpi.2024.100405 39687668 PMC11648780

[46] Abhishek A Jha RK Sinha R Jha K. Automated detection and classification of leukemia on a subject-independent test dataset using deep transfer learning supported by grad-CAM visualization. Biomed Signal Process Control. (2023) 83:104722. 10.1016/j.bspc.2023.104722

[47] Abbas A Abdulrazaq MB Adel AZ. Efficient lightweight CNN for automated classification of B-cell acute lymphoblastic leukemia. Comput Biol Chem. (2025) 120:108645. 10.1016/j.compbiolchem.2025.108645 40845691

[48] Wang Z Xiao J Li J Li H Wang L. WBC-AMNet: automatic classification of WBC images using deep feature fusion network based on focalized attention mechanism. PLoS One. (2022) 17(1):e0261848. 10.1371/journal.pone.0261848 35085275 PMC8794158

[49] Bhatia K Dhalla S Mittal A Gupta S Gupta A Jindal A. Integrating explainability into deep learning-based models for white blood cells classification. Computers & Electr Eng. (2023) 110:108913. 10.1016/j.compeleceng.2023.108913

[50] Reena R Ojha V Debnath B Samarwar SK Vijay SA Sharma N. Explainable AI for leukemia diagnosis: interpreting optimized deep learning models for improved clinical decision-making. In: 2025 IEEE International Conference on Emerging Technologies and Applications (MPSec ICETA). Piscataway, NJ: IEEE (2025). p. 1–6.

[51] Howard A Sandler M Chu G Chen LC Chen B Tan M. Searching for mobilenetv3. In: Proceedings of the IEEE/CVF International conference on Computer vision (2019). p. 1314–24. 10.1109/ICCV.2019.00140

[52] Tan M Le Q. Efficientnet: rethinking model scaling for convolutional neural networks. In: Chaudhuri K, Salakhutdinov R, editors. Proc. 36th Int. Conf. Mach. Learn. PMLR, vol. 97. (2019).

[53] Ma N Zhang X Zheng HT Sun J. Shufflenet v2: practical guidelines for efficient cnn architecture design. In: Ferrari V, Hebert M, Sminchisescu C, Weiss Y, editors. Computer Vision – ECCV 2018. Lecture Notes in Computer Science, vol. 11218. Cham: Springer International Publishing (2018). p. 116–31.

[54] Guo C Pleiss G Sun Y Weinberger KQ. On calibration of modern neural networks. In: Precup D, Teh YW, editors. Proc. 34th Int. Conf. Mach. Learn. PMLR, vol. 70. (2017).

[55] Nixon J Dusenberry MW Zhang L Jerfel G Tran D. Measuring calibration in deep learning. In: Proceedings of the IEEE/CVF Conference on Computer Vision and Pattern Recognition (CVPR) Workshops (2019). p. 38–41.

[56] Vovk V Gammerman A Shafer G. Algorithmic Learning in a Random world. Boston, MA: Springer US (2005).

[57] Angelopoulos AN Bates S. Conformal prediction: a gentle introduction. Foundations and Trends® in Machine learning. (2023) 16(4):494–591. 10.1561/2200000101

[58] Sharvani R Sahu B Singh P. White blood cell classification using deep transfer learning. In: Sing BK, Sinha GR, editors. International Conference on Biomedical Engineering Science and Technology. Cham: Springer Nature Switzerland (2023). p. 220–32.

[59] Błasiok J Gopalan P Hu L Nakkiran P. A unifying theory of distance from calibration. In: Saha B, Servedio RA, editors. Proceedings of the 55th Annual ACM Symposium on Theory of Computing. New York, NY: Association for Computing Machinery (2023). p. 1727–40.

[60] Famiglini L Campagner A Cabitza F. Towards a rigorous calibration assessment framework: advancements in metrics, methods, and use. Front Artif Intell Appl. (2023) 372:645–52. 10.3233/FAIA230327

[61] DeGroot MH Fienberg SE. The comparison and evaluation of forecasters. J R Stat Soc Ser D. (1983) 32(1-2):12–22. 10.2307/2987588

[62] Błasiok J Nakkiran P. Smooth ECE: Principled reliability diagrams via kernel smoothing. arXiv preprint arXiv:2309.12236 (2023).

