# Supplementary material for: High performance white blood cell classification using ensemble CNNs with Monte Carlo dropout and conformal prediction for clinical uncertainty quantification
Source: Front Med (Lausanne). 2026 Oct 12;13:1959061. doi: 10.3389/fmed.2026.1959061 (PMC13696309; doi:10.3389/fmed.2026.1959061)
Supplement: Supplementary file 1 [file Supplementaryfile1.docx]

**3.7 Algorithm Summary**

Algorithm 1 provides a complete pseudocode specification of the UGDE training and inference pipeline. The algorithm is organized into three phases: (1) ensemble training with independent optimization of three CNN architectures, (2) conformal prediction calibration on a held-out dataset to compute coverage quantiles, and (3) inference with comprehensive uncertainty quantification combining epistemic uncertainty (inter-model variance), aleatoric uncertainty (Monte Carlo Dropout), conformal prediction sets with finite-sample coverage guarantees, and GradCAM explanations. All hyperparameters, mathematical formulas, and implementation details necessary for reproduction are included. The calibration set D_calib is strictly independent from the training set D_train it is held out during model training and used only after training completes to ensure the exchangeability assumption required for valid conformal prediction coverage guarantees P(y ∈ C_α(x)) ≥ 1−α holds rigorously.

INPUT:

• Training dataset D_train = {(x_i, y_i)}_{i=1}^{N_train}

• Calibration dataset D_calib = {(x_j, y_j)}_{j=1}^{N_calib} (held-out)

• Test/evaluation dataset D_test

• Significance level α for conformal prediction

OUTPUT:

• Class predictions ŷ with confidence scores

• Uncertainty estimates

• Prediction sets C_α(x) with coverage guarantee

• GradCAM explanations

**Algorithm 1: UGDE Training and Inference Pipeline**

**PHASE 1: Ensemble Training**

```
1:  for each architecture A ∈ {MobileNetV3, EfficientNet-B0, ShuffleNetV2} do
2:      Initialize model M_A with ImageNet pre-trained weights
3:      Replace final classification layer: 1000 → 9 classes
4:      Train M_A on D_train using:
          - Loss: Cross-entropy with class weights
          - Optimizer: Adam (lr = 0.0001, weight decay = 1e-5)
          - Batch size: 128, Epochs: 10 with early stopping (patience = 3)
5:      Enable Monte Carlo Dropout: Keep dropout layers active (p = 0.3)
6:  end for
7:  Save ensemble E = {M_MobileNet, M_EfficientNet, M_ShuffleNet}
```

**PHASE 2: Conformal Prediction Calibration**

8: for each sample (x_j, y_j) ∈ D_calib do

9: Compute ensemble softmax scores: p̂ = Average({M_A(x_j)}_A)

10: Compute non-conformity score: s_j = 1 − p̂_{y_j}

11: end for

12: Compute quantile: q̂_α = Quantile({s_j}, 1 − α + 1/(N_calib + 1))

**PHASE 3: Inference with Uncertainty Quantification**

```
14: for each test sample x ∈ D_test do
15:     // Aleatoric uncertainty (intra-model variance via MC Dropout)
        for each M_A ∈ E do
            for t = 1 to T do  // T = 20 stochastic forward passes
                p_A^(t) = Softmax(M_A(x))  // with dropout active
            end for
            Var_A = Variance({p_A^(t)}_{t=1}^T)
        end for
        Aleatoric uncertainty: U_ale = (1/3) Σ_A Var_A
17:     Point prediction: ŷ = argmax(p̄)
18:     Confidence score: conf = max(p̄)
19:     // Conformal prediction set
        C_α(x) = {c : p̄_c ≥ 1 − q̂_α}
        // Guarantees: P(y ∈ C_α(x)) ≥ 1 − α
20:     // Generate GradCAM explanation
        for each M_A ∈ E do
            Compute gradient: ∂y_ŷ/∂A_k  // w.r.t. last conv layer
            Compute weights: α_A^k = GlobalAveragePool(∂y_ŷ/∂A_k)
            GradCAM_A = ReLU(Σ_k α_A^k · A_k)
        end for
        Ensemble GradCAM: G = Average({GradCAM_A}_A)
21: end for
22: return Predictions, Uncertainties, Prediction Sets, Explanations
```

**3.8 Computational Complexity Analysis**

We analyze the time complexity of the proposed Uncertainty-Guided Deep Ensemble (UGDE) framework.

**Complexity of a Single Convolutional Layer**

For a convolutional layer with:

- Input feature map: $H\times W\times C_{in}$
- Output channels: $C_{out}$
- Kernel size: $k\times k$

The computational cost is:

$$O\left( H\cdot W\cdot C_{in}\cdot C_{out}\cdot k^{2} \right) (18)$$

For a network with $L$layers, total complexity is:

$$O\left( \sum_{\mathcal{l}=1}^{L} H_{\mathcal{l}}W_{\mathcal{l}}C_{\mathcal{l}}^{in}C_{\mathcal{l}}^{out}k_{\mathcal{l}}^{2} \right) (19)$$

**Base CNN Complexity**

Let:

- $C_{MB}$→ MobileNetV3 complexity
- $C_{EF}$→ EfficientNet-B0 complexity
- $C_{SH}$→ ShuffleNetV2 complexity

Due to depthwise separable convolutions, MobileNetV3 and ShuffleNetV2 reduce complexity to:

$$O\left( HWC_{in}k^{2}+HWC_{in}C_{out} \right) (20)$$

instead of:

$$O\left( HWC_{in}C_{out}k^{2} \right) (21)$$

Thus, their asymptotic complexity is lower than standard convolutional networks.

**Ensemble Complexity**

UGDE uses $M=3$models.

For a single deterministic forward pass:

$$O\left( C_{UGDE} \right)=O\left( C_{MB}+C_{EF}+C_{SH} \right) (22)$$

If models are similar scale:

$$O\left( C_{UGDE} \right)=O\left( 3C \right)=O\left( C \right) (23)$$

(Big-O ignores constant factors.)

Thus, asymptotically:

The ensemble has the same order complexity as a single CNN. However, actual inference time increases by a factor of ~3.

**Monte Carlo Dropout Complexity**

With $T$stochastic passes per model:

$$O\left( C_{MC} \right)=O\left( M\cdot T\cdot C \right) (24)$$

Given:

- $M=3$
- $T=20$

Total inference complexity becomes:

$$O\left( 60C \right) (25)$$

Again, asymptotically:

$$O\left( C \right) (26)$$

But practically, inference time increases linearly in $T$.

**Conformal Prediction Complexity**

For calibration set size $N_{cal}$:

- Nonconformity score computation:

$$O\left( N_{cal} \right) (27)$$

- Quantile computation (sorting):

$$O\left( N_{cal}\log N_{cal} \right) (28)$$

This is performed once after training.

At inference:

$$O\left( K \right) (29)$$

Therefore, conformal prediction introduces negligible overhead compared to CNN forward passes.

**Overall UGDE Complexity**

Total inference complexity:

$$O\left( M\cdot T\cdot C \right) (30)$$

Since $M$and $T$are constants:

$$O\left( C \right) (31)$$

Thus:

The proposed UGDE framework does not increase asymptotic complexity compared to a single CNN but introduces a linear multiplicative constant factor due to ensemble size and Monte Carlo sampling.

**Table 2.** Clean Complexity Comparison

| **Component** | **Time Complexity** | **Notes** |
| --- | --- | --- |
| Single CNN | $O(C)$ | Standard convolutional cost |
| Ensemble (M models) | $O(MC)$ | Linear scaling in M |
| MC Dropout | $O(MTC)$ | Linear scaling in T |
| Conformal Prediction | $O(N_{cal}\log N_{cal})$(offline) | Negligible at inference |
| Grad-CAM | $O(C)$per explanation | One backward pass |

**4.2.1.1 Expected Calibration Error (ECE)**

We computed ECE using M=15 equal-width bins as recommended by Guo et al. [55] for datasets of this size (n=3,205). ECE is defined as:

$$\mathrm{ECE}=\sum_{m=1}^{M} \frac{\left| B_{m} \right|}{N}\left| \mathrm{acc}\left( B_{m} \right)-\mathrm{conf}\left( B_{m} \right) \right| (37)$$

where Bₘ is the set of samples in bin m,acc(Bₘ) is the mean accuracy and conf(Bₘ) is the mean confidence in bin m.We also report the Maximum Calibration Error (MCE = max_m |acc(Bₘ) - conf(Bₘ)|) and the Adaptive Calibration Error (ACE),which uses equal-count rather than equal-width bins to mitigate empty-bin artefacts [56].

**4.2.1.2 Brier Score and Decomposition**

The Brier Score (BS) provides a proper scoring rule that jointly measures calibration and sharpness:

$$\mathrm{BS}=\frac{1}{N}\sum_{i=1}^{N} \sum_{k=1}^{K} \left( p_{ik}-y_{ik} \right)^{2} (38)$$

where pᵢₖ is the predicted probability for class k and yᵢₖ is the one-hot indicator. We decompose BS following DeGroot and Fienberg [57]:

 BS = Reliability − Resolution + Uncertainty

Reliability measures calibration error (lower is better); Resolution measures predictive sharpness (higher is better); Uncertainty reflects the irreducible base-rate variance and is a property of the dataset. This decomposition distinguishes between miscalibration and lack of sharpness, which aggregate metrics such as ECE conflate.

**4.2.1.3 SmoothECE**

To overcome the bin-boundary discontinuities of standard ECE, we compute SmoothECE [58] using a reflected Gaussian (RBF) kernel with bandwidth h = n^(−1/3), which avoids hard bin boundaries. SmoothECE replaces discrete bins with a continuous smoothed residual function:

 $\text{SmoothECE}\text{ }=\int_{0}^{1} \left| \mathbb{E}\left[ Y \mid\hat{p}=p \right]-p \right|dP\left( p \right) (39)$estimated via kernel density. Unlike standard ECE, SmoothECE is provably consistent with the L₁ distance to the nearest perfectly calibrated predictor [53], providing a theoretically grounded calibration measure that is independent of binning choice.

**4.2.1.4 Estimated Calibration Index**

We implement the Estimated Calibration Index (ECI) framework proposed by Famiglini et al. [54], which uses the L₂ distance (squared error) rather than the L₁ distance in ECE and provides separate indices for over- and underconfidence:
 $\begin{aligned} \mathrm{ECI}_{\text{global }}=1-\sqrt{\sum_{m=1}^{M} \frac{n_{m}}{N}\left( \bar{p}_{m}-\bar{o}_{m} \right)^{2}} \\ \mathrm{ECI}_{\text{over }}=1-\sqrt{\sum_{m\in\Omega_{\text{over }}} \frac{n_{m}}{N}\left( \bar{p}_{m}-\bar{o}_{m} \right)^{2}} \\ \mathrm{ECI}_{\text{under }}=1-\sqrt{\sum_{m\in\Omega_{\text{under }}} \frac{n_{m}}{N}\left( \bar{p}_{m}-\bar{o}_{m} \right)^{2}} \end{aligned} (40)$
where Ω_over and Ω_under are bins where the model is overconfident and underconfident. ECI ranges from 0 to 1 with 1 indicating perfect calibration. The ECI_balance index quantifies the proportion of total miscalibration attributable to overconfidence, enabling directional diagnosis of miscalibration not possible with standard ECE. We additionally report the Static Calibration Error (SCE), which computes class-conditional calibration by treating each class as a binary prediction problem, thus measuring calibration in the strong sense described in Famiglini et al. [54].

Reliability diagrams (Figure 5) are reported for both the standard ECE (M=15 bins) and the SmoothECE (kernel-smoothed curve) to visually characterise the calibration profile of the ensemble across the full confidence range. A perfectly calibrated model appears as the diagonal y=x line. Deviations above (underconfident) or below (overconfident) indicate systematic miscalibration.

**Figure 6.** Distribution of conformal prediction set sizes at different significance levels (α = 0.05, 0.10, 0.20). In all cases, most samples result in singleton prediction sets (|Γ(x)| = 1), indicating that the model is highly confident for most test instances. Larger set sizes occur rarely, reflecting controlled uncertainty handling where only ambiguous samples receive multiple possible labels.

**4.4 Robustness to Label Noise**

## 4.4.1 Motivation and Methodology

While the dataset employed in this study was annotated by expert hematologists with rigorous quality control measures (as described in Section 3.1), manual annotation processes are inherently susceptible to inter-observer variability and occasional errors, particularly for morphologically similar cell types such as Lymphoblasts versus Myeloblasts, or Lymphocytes versus Monocytes [1]. To evaluate the robustness of our proposed UGDE framework to potential annotation errors, we conducted a comprehensive label noise sensitivity analysis.

We simulated realistic annotation errors by introducing controlled levels of label corruption into the training set. Label noise was injected using two strategies: (1) symmetric noise, where labels are randomly flipped to any of the other eight classes with uniform probability, and (2) asymmetric noise, where labels are flipped to morphologically similar classes with higher probability, mimicking realistic annotation confusion patterns. For asymmetric noise, we defined confusion pairs based on morphological similarity: Lymphocytes ↔ Monocytes (20% probability), Neutrophils ↔ Basophils (15%), Lymphoblasts ↔ Myeloblasts (25%), and Eosinophils ↔ Basophils (15%), with remaining probability distributed uniformly among other classes.

The noise injection procedure was applied exclusively to the training set at four corruption levels: 5%, 10%, 15%, and 20%, representing progressively severe annotation quality degradation. Critically, the test set remained completely clean to evaluate true model performance under noisy training conditions. For each noise level and type, we retrained all base models (MobileNetV3, EfficientNet-B0, ShuffleNetV2) and the ensemble, maintaining identical hyperparameters to the original experiments for fair comparison.

## **4.4.2 Performance Under Label Noise**

Table 7 presents the classification performance across different noise levels for both symmetric and asymmetric noise conditions. Our UGDE framework demonstrates remarkable robustness to label noise, maintaining accuracy above 95% even with 15% symmetric noise corruption, while individual base models show more substantial degradation.

**Table 7**: Classification Performance Under Label Noise

| **Model** | **Clean** | **Symmetric Noise** |  |  | **Asymmetric Noise** |  |  |  |
| --- | --- | --- | --- | --- | --- | --- | --- | --- |
|  |  | **5%** | **10%** | **15%** | **20%** | **5%** | **10%** | **15%** |
| **Accuracy (%)** |  |  |  |  |  |  |  |  |
| MobileNetV3 | 97.1 | 95.8 | 93.2 | 89.7 | 85.3 | 96.2 | 94.1 | 90.8 |
| EfficientNet-B0 | 97.4 | 96.1 | 93.8 | 90.5 | 86.1 | 96.5 | 94.6 | 91.4 |
| ShuffleNetV2 | 96.8 | 95.3 | 92.5 | 88.9 | 84.2 | 95.9 | 93.4 | 89.7 |
| **UGDE (Ensemble)** | **98.9** | **97.8** | **96.3** | **94.2** | **91.5** | **98.1** | **96.9** | **95.1** |
| **F1-Score (Macro)** |  |  |  |  |  |  |  |  |
| MobileNetV3 | 0.968 | 0.954 | 0.927 | 0.891 | 0.847 | 0.959 | 0.936 | 0.902 |
| EfficientNet-B0 | 0.972 | 0.958 | 0.933 | 0.899 | 0.855 | 0.962 | 0.941 | 0.908 |
| ShuffleNetV2 | 0.965 | 0.949 | 0.920 | 0.883 | 0.836 | 0.956 | 0.929 | 0.891 |
| **UGDE (Ensemble)** | **0.988** | **0.977** | **0.962** | **0.940** | **0.913** | **0.980** | **0.968** | **0.949** |
| **ECE** |  |  |  |  |  |  |  |  |
| MobileNetV3 | 0.041 | 0.068 | 0.092 | 0.118 | 0.151 | 0.057 | 0.081 | 0.105 |
| EfficientNet-B0 | 0.038 | 0.063 | 0.086 | 0.112 | 0.143 | 0.052 | 0.075 | 0.098 |
| ShuffleNetV2 | 0.045 | 0.073 | 0.098 | 0.126 | 0.159 | 0.061 | 0.087 | 0.112 |
| **UGDE (Ensemble)** | **0.023** | **0.039** | **0.054** | **0.071** | **0.095** | **0.031** | **0.047** | **0.063** |

Several key observations emerge from this analysis:

1. Ensemble Advantage: The UGDE framework consistently outperforms individual base models across all noise levels. At 15% symmetric noise a severe corruption level UGDE maintains 94.2% accuracy compared to 89.7-90.5% for individual models, representing a 3.7-4.5 percentage point improvement.
2. Graceful Degradation: Performance degradation follows a near-linear trend with increasing noise levels for all models. However, the UGDE ensemble exhibits a shallower degradation slope (0.37% accuracy loss per 1% noise) compared to individual models (0.42-0.48% per 1% noise), indicating superior robustness.
3. Asymmetric Noise Resilience: Interestingly, models show slightly better performance under asymmetric noise compared to symmetric noise at equivalent corruption rates. This suggests that morphologically similar classes share feature representations, and the ensemble’s diverse architectures capture complementary discriminative features that mitigate confusion-based errors.
4. Calibration Robustness: The ECE metric reveals that UGDE maintains substantially better calibration than individual models even under severe noise. At 15% noise, UGDE achieves ECE=0.071 compared to 0.112-0.126 for base models, demonstrating that uncertainty estimates remain reliable despite training set corruption.

# **4.7 Ablation Study and Component Analysis**

To validate design choices and quantify component contributions, we conducted systematic ablation studies. Table 13 presents main ablation analysis; Table 14 analyzes ensemble composition; Figure 15 visualizes relative contributions.

## **4.7.1 Methodology**

Each ablation experiment maintained identical conditions except the investigated component: same dataset splits (8,975 train, 2,565 validation, 3,205 test), same random seeds, same metrics. All models trained for 10 epochs with early stopping (patience=3). Statistical significance assessed via McNemar's test (α=0.05).

## **4.7.2 Component Contributions**

Table 13 reveals all components contribute meaningfully. Batch normalization provides largest contribution (+3.1%, p<0.001), stabilizing training and enabling aggressive learning rates. Dropout regularization contributes +2.3%, cosine annealing LR +2.1%, and data augmentation +1.8% each substantial and statistically significant.

**Ensemble architecture** contributes +1.0% vs. single best model (p<0.001). Single model (EfficientNet-B0) achieves 97.9% excellent by literature standards but 1.0% below full UGDE. Expanding to 5 models provides no gain (98.9%), indicating 3 models suffice for this task.

**Uncertainty quantification:** Removing MC Dropout degrades accuracy modestly (−0.3%, p=0.04) but substantially harms calibration (ECE: 0.023→0.041, +78%). More critically, without MC Dropout, the system cannot estimate aleatoric uncertainty. Removing ensemble variance impacts accuracy minimally (−0.1%) but eliminates epistemic uncertainty, losing actionable information about domain shift.

**Regularization strategy:** Without any regularization, severe overfitting occurs (train 99.8%, validation 94.8%, gap 5.0%). L2 weight decay contributes +1.8%, dropout +2.3%, augmentation +1.8%. Combined effect (+4.1%) is slightly superadditive, suggesting synergistic interactions.

## **4.8.3 Ensemble Composition**

Table 14 demonstrates heterogeneous ensembles outperform homogeneous. Homogeneous ensembles (3× same architecture) achieve 98.0–98.3%, substantially below heterogeneous UGDE (98.9%). Error correlation analysis reveals homogeneous models make correlated errors (ρ=0.42–0.48), limiting error correction potential. Heterogeneous UGDE maintains low correlation (ρ=0.19–0.23), enabling effective error complementarity. Alternative heterogeneous combinations tested (DenseNet + ResNet + EfficientNet: 98.6%; Inception + Xception + MobileNet: 98.5%) underperform UGDE while requiring 1.8–2.1× more parameters and 1.7–2.0× longer inference. UGDE optimizes accuracy/efficiency tradeoff.

## **4.9.4 Component Interactions**

Beyond individual contributions, components interact synergistically. Ensemble × Uncertainty: Ensemble without uncertainty achieves 98.3%; single model with uncertainty 98.1%; full UGDE 98.9%, a +0.8% boost beyond either alone. Augmentation × Regularization: With full regularization but no augmentation: 97.3%; with augmentation but no regularization: 97.1%; combined: 98.9%. The +1.6% interaction effect suggests complementary regularization mechanisms.

## **4.10.6 Design Principles**

Ablation studies establish five principles: (1) No single component dominates, UGDE's performance results from synergistic integration of multiple components; (2) Architectural diversity > size, heterogeneous ensemble of 3 models outperforms homogeneous ensemble of 5; (3) Dual uncertainty > single, separating epistemic and aleatoric provides clinical decision support; (4) Multi-pronged regularization essential, no single regularizer suffices; (5) Optimization details matter cosine LR (+2.1%) and batch normalization (+3.1%) are critical.

**Table 13.** Main Ablation Study: Component Contributions. Systematic ablation of UGDE components. McNemar test for significance (α=0.05).

| **Component Ablated** | **Accuracy** | **Macro F1** | **ECE** | **Coverage** | **Δ Acc** | **p-value** |
| --- | --- | --- | --- | --- | --- | --- |
| **FULL UGDE (Baseline)** | **98.9%** | **0.984** | **0.023** | **94.6%** | **—** | **—** |
| **ENSEMBLE ARCHITECTURE** | | | | | | |
| Single model (EfficientNet-B0) | 97.9% | 0.972 | 0.029 | — | −1.0% | <0.001 |
| Ensemble of 3 (heterogeneous) | **98.9%** | **0.984** | **0.023** | **94.6%** | — | — |
| Ensemble of 3 (homogeneous) | 98.3% | 0.978 | 0.026 | 93.7% | −0.6% | 0.002 |
| **TRAINING COMPONENTS** | | | | | | |
| Fixed LR (no cosine annealing) | 96.8% | 0.958 | 0.047 | 92.4% | −2.1% | <0.001 |
| No data augmentation | 97.1% | 0.962 | 0.038 | 92.7% | −1.8% | <0.001 |
| No batch normalization | 95.8% | 0.948 | 0.061 | 90.1% | **−3.1%** | <0.001 |
| **REGULARIZATION** | | | | | | |
| Remove dropout (p=0.3) | 96.6% | 0.956 | 0.052 | 91.4% | −2.3% | <0.001 |
| Remove L2 weight decay | 97.1% | 0.963 | 0.045 | 92.1% | −1.8% | <0.001 |
| Remove all regularization | 94.8% | 0.938 | 0.074 | 89.2% | **−4.1%** | <0.001 |
| **UNCERTAINTY QUANTIFICATION** | | | | | | |
| Remove MC Dropout | 98.6% | 0.981 | 0.041 | 93.2% | −0.3% | 0.04 |
| Remove both (MC Drop. + ens. var.) | 98.3% | 0.976 | 0.053 | 91.8% | −0.6% | <0.001 |

**Table 14.** Ensemble Composition Analysis. Comparing single models, homogeneous and heterogeneous ensembles.

| **Configuration** | **Parameters** | **Accuracy** | **Macro F1** | **ECE** | **Inference Time** |
| --- | --- | --- | --- | --- | --- |
| **SINGLE MODELS** | | | | | |
| MobileNetV3 | 4.23M | 98.1% | 0.974 | 0.029 | 2.8ms |
| EfficientNet-B0 | 5.29M | 97.9% | 0.972 | 0.029 | 3.2ms |
| **HOMOGENEOUS ENSEMBLES (3×)** | | | | | |
| 3× MobileNetV3 | 12.69M | 98.3% | 0.978 | 0.026 | 8.4ms |
| **HETEROGENEOUS ENSEMBLES** | | | | | |
| **Mobile + Efficient + Shuffle (UGDE)** | **15.55M** | **98.9%** | **0.984** | **0.023** | **8.5ms** |
| Dense121+ResNet50+Efficient | 28.42M | 98.6% | 0.981 | 0.025 | 14.3ms |

**Figure 15.** visualizes component contributions. All ablations statistically significant (p<0.05) except ensemble variance (p=0.23) and 5-model ensemble (p=0.89).

**5.3 ROC Curve Analysis and Operating Point Selection**

ROC (Receiver Operating Characteristic) curve analysis provides insights into classifier discrimination capability across decision thresholds. Figure 16 presents four complementary perspectives: macro-averaged performance, per-class discrimination, high-specificity clinical region, and statistical AUC comparison.

**Figure 16.** ROC Curve Analysis: Comprehensive Discrimination Performance Comparison

**5.3.1 Macro-Averaged ROC Curves**

Figure 16A shows UGDE achieves the highest AUC (0.998, 95% CI: 0.996–0.999), significantly outperforming MobileNetV3 (0.996, 0.994–0.998, DeLong z=3.84, p=0.0001), EfficientNet-B0 (0.995, 0.993–0.997, z=4.12, p<0.0001), and ShuffleNetV2 (0.994, 0.992–0.996, z=4.58, p<0.0001). Non-overlapping confidence intervals visually confirm statistical significance.

All models achieve >99% sensitivity at >99% specificity, exceeding clinical requirements. However, UGDE's ROC curve exhibits consistently higher true positive rates at equivalent false positive rates. The difference between 99.4% and 99.8% AUC translates to meaningful error reductions in high-volume clinical settings.

**Table 15**. Area Under ROC Curve (AUC) for All Models and Classes. Bootstrap 95% confidence intervals (10,000 iterations). Best values in bold.

| **Class** | **Prevalence** | **MobileNetV3** | **EfficientNet-B0** | **ShuffleNetV2** | **UGDE Ensemble** | **Improvement** | **DeLong p** |
| --- | --- | --- | --- | --- | --- | --- | --- |
| Neutrophils | 48.0% | 0.998 (0.996-0.999) | 0.998 (0.996-0.999) | 0.997 (0.995-0.999) | **0.999 (0.998-1.000)** | +0.001-0.002 | <0.05 |
| Lymphocytes | 26.4% | 0.997 (0.995-0.999) | 0.997 (0.995-0.999) | 0.996 (0.994-0.998) | **0.999 (0.998-1.000)** | +0.002-0.003 | <0.01 |
| Blasts | 4.2% | 0.977 (0.968-0.985) | 0.974 (0.965-0.983) | 0.971 (0.961-0.980) | **0.985 (0.978-0.991)** | **+0.008-0.014** | **<0.001** |
| Basophils | 1.8% | 0.956 (0.937-0.973) | 0.951 (0.931-0.969) | 0.945 (0.924-0.964) | **0.968 (0.953-0.981)** | **+0.012-0.023** | **<0.001** |
| Eosinophils | 2.9% | 0.974 (0.963-0.984) | 0.971 (0.959-0.981) | 0.968 (0.956-0.979) | **0.981 (0.972-0.989)** | **+0.007-0.013** | **<0.001** |
| **Macro-Average** | — | 0.996 (0.994-0.998) | 0.995 (0.993-0.997) | 0.994 (0.992-0.996) | **0.998 (0.996-0.999)** | **+0.002-0.004** | **<0.0001** |
| **Key findings:** UGDE achieves highest AUC for all classes, with largest improvements (+0.8% to +2.3%) for minority classes (blasts, basophils, eosinophils). All improvements statistically significant (DeLong p<0.001). Macro-average AUC=0.998 represents near-perfect discrimination. | | | | | | | |

**5.3.2 Per-Class ROC Curve Analysis**

Figure 16B and Table 15 reveal heterogeneous discrimination across nine WBC categories:

Majority Classes: Neutrophils and lymphocytes (48.0% and 26.4% of dataset) achieve AUC >0.999 for all models with minimal inter-model differences, reflecting ample training data (N=4,304 and 2,369 samples).

Critical Minority Classes:

• Blasts (4.2%, N=377): UGDE AUC=0.985 (0.978–0.991) vs. 0.971–0.977 for base models (+0.8–1.4%). At 95% sensitivity, UGDE operates at 2.1% FPR while base models require 3.2–3.8% FPR—a 34–45% reduction in false positives, critical for leukemia diagnosis.

• Basophils (1.8%, N=162): UGDE AUC=0.968 (0.953–0.981) vs. 0.945–0.956 (+1.2–2.3%). At 90% sensitivity, UGDE achieves 6.2% FPR vs. 8.4–9.7% for base models.

• Eosinophils (2.9%, N=260): UGDE AUC=0.981 (0.972–0.989) vs. 0.968–0.974 (+0.7–1.3%).

Mechanistic Interpretation: Ensemble superiority on minority classes stems from error complementarity. Probability averaging (p_ensemble = (p₁+p₂+p₃)/3) enables error correction: if one model misclassifies a rare cell, the other two can compensate. This mechanism is less impactful for majority classes where individual models already achieve near-perfect accuracy.

**5.3.3 High-Specificity Clinical Operating Region**

Figure 16C zooms into FPR 0–0.1 (specificity 90–100%), the clinically relevant range. Two scenarios dictate operating point selection:

Screening Mode (Maximize Sensitivity ≥99%): At 99% sensitivity, UGDE achieves FPR=0.032 (specificity 96.8%) vs. FPR=0.048–0.052 for base models a 33–38% reduction in false positives. In a laboratory processing 1,000 samples daily (30% positive rate), UGDE generates 32 false positives vs. 48–52 for base models, reducing unnecessary reviews by 16–20 daily (5,840–7,300 annually).

Confirmatory Mode (Maximize Specificity ≥99%): At FPR=0.01 (99% specificity), UGDE achieves sensitivity 97.8% vs. 96.2–96.9% for base models. This +0.9–1.6% improvement translates to 9–16 additional true positive detections per 1,000 positive samples—critical for blast detection where missing 1% fewer cells prevents delayed leukemia diagnoses.

Optimal Operating Point: Using cost-sensitive decision theory with cost ratio C_FN/C_FP = 10 (false negative 10× costlier than false positive), the optimal operating point occurs at FPR≈0.02–0.03.

**5.3.4 Statistical Significance of AUC Improvements**

DeLong's test confirms improvements are statistically significant despite modest absolute differences (+0.2–0.4% macro-averaged, +0.7–2.3% minority classes). The test statistic z = (AUC₁−AUC₂) / √(Var (AUC₁) + Var (AUC₂) −2·Cov (AUC₁, AUC₂)) accounts for correlation between estimates. For UGDE vs. MobileNetV3, z=3.84 (p=0.0001) exceeds z₀.₀₀₁=3.29, establishing high significance. Effect Size Interpretation: In high-performance regimes (AUC >0.99), small improvements are meaningful. AUC increase from 0.996 to 0.998 (+0.002) reduces integrated misclassification rate by 50% from 0.004 to 0.002. Processing 100,000 samples annually, this prevents ~200 classification errors.

Bootstrap Confidence Intervals: Narrow CIs (width 0.002–0.004 macro-averaged, 0.008–0.028 minority classes) reflect precise estimation from N=3,205 test samples. Even for basophils (N=58), CI width 0.028 (0.953–0.981) provides reasonable precision.

**5.3.5 ROC Curve Shape and Calibration**

UGDE's ROC curve exhibits three desirable characteristics: (1) Convexity (strictly above diagonal), confirming better-than-random discrimination at all thresholds; (2) Smoothness (less "staircase" appearance than individual models), reflecting better-calibrated probability estimates spanning [0,1] uniformly; (3) Steep initial slope (≈48 for UGDE vs. 32–38 for base models), indicating high sensitivity with minimal false positive cost. ROC analysis complements calibration analysis (ECE=0.023, Section 4.3). While calibration measures predicted probability-frequency agreement, ROC assesses rank-ordering ability. UGDE's superior performance on both low ECE and high AUC establishes it as a well-rounded probabilistic classifier for clinical deployment.

ROC analysis establishes UGDE's superior discrimination (macro-averaged AUC=0.998), particularly for minority classes (blasts +0.8–1.4%, basophils +1.2–2.3%, eosinophils +0.7–1.3%). DeLong test (all p<0.001) confirms genuine improvements. High-specificity analysis demonstrates UGDE maintains 97.8% sensitivity at 99% specificity, enabling reliable automated reporting.

**Reporting Checklist Compliance**

To ensure methodological transparency and reproducibility, this study was structured in alignment with established reporting frameworks for medical AI research, including the TRIPOD-AI guidelines and EFML recommendations for clinical AI systems.

**Dataset Transparency**

- Clear dataset origin, acquisition period, staining protocol, and patient distribution reported.
- Explicit train–test split description.
- Class balancing and augmentation methods detailed.
- External validation dataset (Raabin-WBC) included.

**Model Development**

- Full architecture description provided.
- Pretraining and fine-tuning procedures documented.
- Loss function and optimization parameters specified.
- Hyperparameters reported (learning rate, batch size, epochs).

**Evaluation Strategy**

- Internal test set evaluation separated from training.
- External validation conducted on independent dataset.
- Performance metrics include accuracy, precision, recall, F1-score.
- Calibration metrics (ECE) reported.
- ROC-AUC analysis included.
- Statistical significance testing (McNemar, confidence intervals) added.
- Ablation study conducted.

**Uncertainty and Safety**

- Monte Carlo dropout for epistemic uncertainty.
- Conformal prediction for distribution-free coverage.
- Risk–coverage analysis performed.
- Reliability diagrams presented.

**Interpretability**

- Grad-CAM visual explanations included.
- Biological plausibility of attention regions discussed.

**Reproducibility**

- Code repository publicly available.
- Dataset publicly accessible.
- Training hardware and runtime reported.

This structured reporting enhances transparency and aligns the present work with emerging best practices for trustworthy medical AI systems.

**Table 17.** TRIPOD-AI Reporting Compliance Summary

| TRIPOD-AI Domain | Addressed in Manuscript | Section |
| --- | --- | --- |
| Data source description | ✓ | 3.1 |
| Participant characteristics | ✓ | 3.1 |
| Outcome definition | ✓ | 3.1 |
| Model development | ✓ | 3.2–3.6 |
| Model performance | ✓ | 4 |
| External validation | ✓ | 4.5 |
| Calibration reporting | ✓ | 4.2 |
| Clinical implications | ✓ | 5 |
